# Whole-exome sequencing of rectal neuroendocrine tumors

**DOI:** 10.1530/ERC-22-0257

**Published:** 2023-08-02

**Authors:** Yuanliang Li, Yiying Guo, Zixuan Cheng, Chao Tian, Yingying Chen, Ruao Chen, Fuhuan Yu, Yanfen Shi, Fei Su, Shuhua Zhao, Zhizheng Wang, Jie Luo, Huangying Tan

**Affiliations:** 1 Department of Integrative Oncology, China-Japan Friendship Hospital, Beijing University of Chinese Medicine, Beijing, China; 2 Department of Integrative Oncology, China-Japan Friendship Hospital, Chinese Academy of Medical Sciences & Peking Union Medical College, Beijing, China; 3 Department of Pathology, China-Japan Friendship Hospital, Beijing, China; 4 Department of Integrative Oncology, China-Japan Friendship Hospital, Beijing, China; 5 Department of Biological Information Research, HaploX Biotechnology Co., Ltd, Shenzhen, Guangdong, China; 6 Academic Department, HaploX Biotechnology, Co., Ltd, Shenzhen, Guangdong, China

**Keywords:** rectal neuroendocrine tumors, whole-exome sequencing, prognosis, targeted therapy, immune checkpoint blockade

## Abstract

The genetic characteristics of rectal neuroendocrine tumors (R-NETs) were poorly understood. Depicting the genetic characteristics may provide a biological basis for prognosis prediction and novel treatment development. Tissues of 18 R-NET patients were analyzed using whole-exome sequencing. The median tumor mutation burden (TMB) and microsatellite instability (MSI) were 1.15 Muts/MB (range, 0.03–23.28) and 0.36 (range, 0.00–10.97), respectively. Genes involved in P53 signaling, PI3K-AKT signaling, DNA damage repair, WNT signaling, etc. were frequently altered. Higher TMB (*P* = 0.078), higher CNV (*P* = 0.110), somatic mutation of *CCDC168* (*P* = 0.049), *HMCN1* (*P* = 0.040), *MYO10* (*P* = 0.007), and amplification of *ZC3H13* (*P* < 0.001) were associated with shorter OS. Potentially targetable gene alterations (PTGAs) were seen in 72% of the patients. *FGFR1* amplification (22%) was the most common PTGA followed by *BARD1* and *BRCA2* mutation (each 17%). As for gene variations associated with the efficacy of immune checkpoint blockade (ICB), *FAT1* alteration (39%) and *PTEN* depletion (28%) were commonly observed. In conclusion, frequently altered oncogenic pathways might contribute to the development and progression of R-NETs. Gene alterations significantly associated with prognosis might be potential novel targets. Targeted therapy might be a promising strategy as targetable alterations were prevalent in R-NETs. *FAT1* alteration and *PTEN* depletion might be the main genetic alterations influencing the response to ICB besides overall low TMB and MSI in R-NETs.

## Introduction

Neuroendocrine neoplasms (NENs) are a group of rare malignancies with strong heterogeneity, which arise from neuroendocrine cells distributed throughout the body and produce peptide hormone and biogenic amine (Klöppel 2017). NENs can affect all parts of the gastrointestinal tract. Rectal neuroendocrine neoplasms (R-NENs) are the most prevalent NENs and rank second for the incidence rate of gastrointestinal NENs, with a rate of 1.04/100,000 (Dasari *et al.* 2017). According to the differentiation, they can be classified as well-differentiated rectal neuroendocrine tumors (R-NETs) and poorly differentiated neuroendocrine carcinomas (R-NECs). In recent years, several drugs or treatment strategies have been developed for the treatment of R-NETs, such as somatostatin analogs (SSAs) (Rinke *et al.* 2009), mTOR inhibitors (Everolimus) (Yao *et al*. 2016), vascular-targeting agents (Surufatinib) (Xu *et al.* 2020), and peptide receptor radionuclide therapy (PRRT) (Strosberg *et al.* 2017). Nevertheless, the treatment options for R-NETs are still limited, especially for advanced patients with drug resistance after a multi-line of treatment. Advanced patients usually suffer from liver metastasis and bear a heavy financial burden with low quality of life. Thus, it is necessary to develop novel drugs for these patients.

So far, the genomic characteristics of R-NETs are not well understood. The current genomic research for NENs mainly focused on the pancreas (Jiao *et al.* 2011, Scarpa *et al.* 2017), small intestine (Banck *et al.* 2013, Francis *et al.* 2013), and lung (Gabriel *et al.* 2020), while the genomic data based on whole-exome sequencing in R-NETs was absent. Evidence has shown that the sensitivity to mTOR inhibitors of R-NETs is higher than that of NETs from other primary sites (Singh *et al.* 2018), suggesting the unique molecular characteristics of R-NETs. However, most of the previous genomic studies have integrated the genomic data of gastroenteropancreatic NENs (Mafficini & Scarpa 2019, Puccini *et al.* 2020, Venizelos *et al.* 2021), while genomic studies analyzing R-NETs alone are rare, which leads to a lack of understanding of the unique genomic characteristics of R-NETs and further limits the drug development for individualized treatment. In this study, we performed whole-exome sequencing for 18 Chinese R-NET patients to comprehensively depict the genetic characteristics based on somatic gene mutation and copy number variation (CNV) in Chinese R-NETs. We investigated frequently altered signaling pathways, variations potentially affecting the prognosis, potentially targetable gene alterations (PTGAs), and gene alterations potentially influencing the efficacy of immune checkpoint blockade (ICB).

## Materials and methods

### Patient selection

Between 2015 and 2021, a total of 66 patients with R-NETs at China-Japan Friendship Hospital in Beijing underwent endoscopic or surgical resection. After excluding patients without sufficient tumor samples or complete clinical/follow-up information, a total of 18 R-NET patients were finally included in this study. Before the commencement of the study, all experimental protocols have been approved by the Ethics Committee of China-Japan Friendship hospital (2019-24-K18-1), and all patients have signed informed consent before collecting tissues or blood. The study was conducted following the declaration of Helsinki.

### Clinicopathological information collection and follow-up

All hematoxylin and eosin-stained tumor sections were examined by gastrointestinal pathologists to confirm the diagnosis and evaluate histopathological features, including tumor size, mitotic count, tumor grade, depth of invasion, lymphatic invasion, vascular invasion, perineural invasion, and status of the resection margin. Clinical information, including age, gender, tumor grade, tumor stage, lymph node involvement, treatment, and overall survival (OS) were collected. The patients were followed up by outpatient visits, ward hospitalization, or telephone inquiry until death occurred or the end of the study in May 2022.

### Sample collection

Eighteen primary R-NET samples including 3 fresh tumor tissues and 15 formalin-fixed paraffin embedding (FFPE) specimens and corresponding blood samples were collected. FFPE or fresh tumor samples were used for whole-exome sequencing with corresponding adjacent normal tissues or peripheral blood lymphocytes used as control. Tumor samples with more than 10% of tumor content were eligible for sequencing, while patients with tumor content of less than 10% were asked for resampling. Two samples were collected after treatment (sample 9 was collected from a patient who had received capecitabine, and sample 12 was from a patient who had received surufatinib). Other samples were all collected before systemic medical treatment.

### Sample processing and whole-exome sequencing

For FFPE samples, genomic DNA was extracted from ten tumor sections of 5 μm using QIAamp DNA FFPE Tissue Kit (Qiagen GmbH). For blood samples, DNA was extracted from peripheral blood lymphocytes (PBL) using a blood genomic DNA Extraction Kit (centrifugal column method) (Tiangen Biotech (Beijing) Co., Ltd). For fresh tumor tissues, DNA was extracted using MagPure FFPE DNA LQ Kit F (Magen Biotechnology Co., Ltd). DNA was quantified using the Qubit 4.0 fluorometer and Qubit dsDNA HS Analysis Kit (Thermo Fisher Scientific, Inc.) according to the manufacturer's instructions. 5× WGS fragmentation mix, 10× WGS fragmentation buffer, T4 DNA Ligase, 5× rapid ligation buffer (Tiangen Biotech (Beijing) Co., Ltd.), KAPA HiFi HotStart Readymix, KAPA Library Amplification Primer Mix (Roche molecular systems, Inc.) were used for library construction. Then, the library was purified by KAPA HyperPure Beads (Roche molecular systems, Inc.). Hybridization capture was carried out using the combined probe of KAPA HyperExplore Max and KAPA HyperExome (Roche molecular systems, Inc.). PCR amplification was carried out for seven to eight cycles according to the input amount of the hybridization library. DNA sequencing was performed on Illumina Novaseq 6000 (Illumina, Inc.) system with an average depth of 650×.

### Genomic data processing and analysis

The Fastp software (v0.23.0) (https://github.com/OpenGene/fastp) was used to filter the raw data. The filtered reads were further aligned with the reference genome hg19 (GRCh37) using BWA MEM (v0.7.15-rll40) (http://bio-bwa.sourceforge.net/). Sambamba (V 0.6.6) (https://github.com/lomereiter/sambamba/issues) was used to remove duplicated results after alignment. The Baserecalibrator program in gatk (v4.1.1.0) was used to calculate the error distribution of base mass. Applybqsr program in gatk (v4.1.1.0) was used to correct the base mass. Mutect2 tool in gatk (v4.1.1.0) was used for somatic mutation (SNVs, Indels) analysis. The default parameter was used for filtering SNVs and Indels. Cnvkit (v0.9.6) was used for CNV analysis (https://cnvkit.readthedocs.io/en/stable/). Further annotation of mutation sites was performed by Annovar (https://annovar.openbioinformatics.org/en/latest/). Msisensor (v0.5) (https://github.com/ding-lab/msisensor) is used for microsatellite instability analysis which counted the number of known microsatellite loci that were altered by somatic insertion or deletion. TMB was calculated by counting the number of nonsynonymous somatic mutations within an average of 1 million bases in the tumor genome. TMB-high (TMB-H) was defined as TMB more than or equal to ten mutations/MB. MSI-high (MSI-H) was defined as the MSI index more than or equal to ten. R package deconstructSigs was used for the analysis of mutational signatures based on the signatures reported by Alexandrov et al. (Alexandrov *et al.* 2013). Mutation and CNV profiles were drawn by the R package Complexheatmap. Five data sources: Cancer Gene Census (https://cancer.sanger.ac.uk/census/), Integrative Onco Genomics (https://www.intogen.org/search), publication by Bert Vogelstein (Vogelstein *et al.* 2013), significantly mutated genes from the The Cancer Genome Atlas (TCGA) Pan-cancer analysis (Kandoth *et al.* 2013), and driver genes derived from integrated analysis (Tamborero *et al.* 2013) were used to screen out known driver genes. Hallmark gene set in Molecular signatures database (Msigdb) and Kyoto Encyclopedia of Genes and Genomes (KEGG) database was used for signaling pathway analysis. The Gene ontology (GO) database was interrogated for functional enrichment analysis by R package clusterProfiler. Potentially targetable gene alterations were interrogated from Oncology Knowledge Base (OncoKB) (https://www.oncokb.org) (Chakravarty *et al.* 2017).

### Statistical analysis and data visualization

Statistical analysis and data visualization were performed using R software (v 4.1.2). For continuous normal distribution variables, the mean ± s.d. was calculated with the Student’s *t*-test applied to show the significance of the difference. For categorical variables, the percentage was calculated with Fisher’s exact test applied to determine the significance of the difference. Survival analysis was performed by R package survival and survminer. The Kaplan–Meier (KM) method was used to estimate the probability of OS, with the log-rank test performed to evaluate significance. All tests were two-sided. *P* < 0.05 was considered statistically significant, while *P* < 0.1 was regarded as a trend.

## Results

### Patient characteristics

Eighteen R-NET patients were included in this study. The clinical information of the included patient is summarized in Table 1. There were 4 females and 14 males. The mean age at the time of sampling was 57.56 (range, 37–78) years (Table 1). There were seven patients in grade 1, ten patients in grade 2, and one patient in grade 3 (Table 1). Most tumors (12/18, 66.67%) had sizes more than 1 cm in the maximum diameters. Six patients (33.3%) had localized or regional disease, while 12 patients (66.7%) presented distant metastasis. Three patients (16.7%) received endoscopy, six patients received (33.3%) radical surgery, and nine patients (50%) received systemic therapy (including one patient treated with capecitabine, five patients with SSAs, three patients with anti-angiogenesis tyrosine kinase inhibitors) as the first treatment, respectively (Table 1).

**Table 1:** Clinical characteristics of 18 RNET patients.

Characteristics		NET
		(*n* = 18)
Sex (%)	Female	4 (22.2)
	Male	14 (77.8)
Age (mean (s.d.))		57.56 (11.55)
Tumor size (median (IQR))		1.70 (1.30, 2.25)
Ki67 (median (IQR))		3.00 (1.25, 8.75)
T stage (%)	T1	6 (33.3)
	T2	3 (16.7)
	T3	0 (0.0)
	T4	4 (22.2)
	Tx	5 (27.8)
N stage (%)	N0	6 (33.3)
	N1	11 (61.1)
	Nx	1 (5.6)
M stage (%)	M0	6 (33.3)
	M1	12 (66.7)
Stage (%)	I	3 (16.7)
	III	3 (16.7)
	IV	12 (66.7)
Grade (%)	G1	7 (38.9)
	G2	10 (55.6)
	G3	1 (5.6)
Treatment (%)	Chemotherapy	1 (5.6)
	Endoscopy	3 (16.7)
	SSA	5 (27.8)
	Surgery	6 (33.3)
	TKI	3 (16.7)

R-NET, rectal neuroendocrine tumor; SSA, somatostatin analog; TKI, tyrosine kinase inhibitor.

### Somatic mutation characteristics

**Figure 1:**
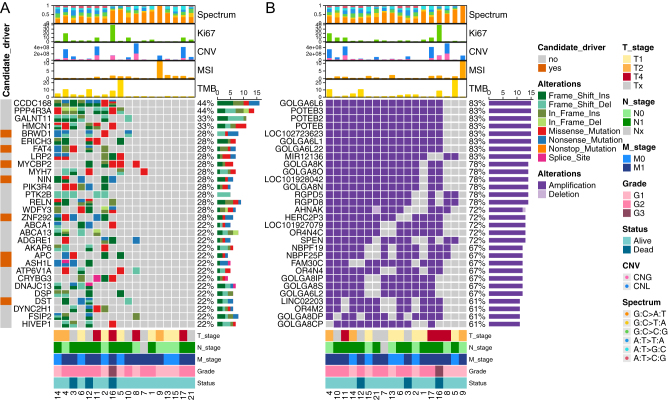
The genomic landscape of the 18 R-NETs shows the top 30 genes with somatic mutation (A) and the top 30 genes with copy number variation (B). Genes were ordered by their frequency in 18 R-NET patients. Clinicopathologic and genetic parameters were annotated in the top bar plots and bottom colored bars. Identified candidate drive genes were annotated in the left colored bar (A). A full-colour version of this figure can be found at https://doi.org/10.1530/ERC-22-0257.

The whole-exome sequencing of 18 R-NETs found a median of 45 single nucleotide variants (SNVs) and 19 small insertion deletions (Indels) for somatic nonsynonymous mutation. Figure 1A showed the top 30 high-frequency gene mutations. *CCDC168* and *PPP4R3A* mutation were the somatic mutations with the highest frequency which were both detected in eight (44.4%) patients. *GALNT11* and *HMCN1* mutations were detected in six (33.3%) patients. Other high-frequency gene mutations included *BRWD1, ERICH3, FAT4, LRP2, MYCBP2, MYH7, NIN, PIK3R4, PTK2B, RELN, WDFY3,* and* ZNF292* (for each gene, *n* = 5, 27.8%). Notably, *APC*, the well-recognized frequently mutated genes in colorectal adenocarcinoma (COREAD) and colorectal NEC (CR-NEC) was mutated in four patients (22.2%) in our R-NET cohort (Fig. 1A). The most common mutational spectrum was G: C>A: T transition (Fig. 1, Supplementary Fig. 1B, see section on supplementary materials given at the end of this article). For mutational signature, signature 1A was the most prevalent (13/18, 72.2%) followed by signature 3 (9/18, 50%) and signature 15 (6/18, 33.3%). Patients were clustered into four groups mainly based on the signature 1A, signature 3, and signature 15. The fourth cluster exhibited few of these three signatures (Supplementary Fig. 1C). All patients had mutations on the chromosome (Chr) 12 and Chr 19 (Supplementary Fig. 1A). For the identification of tumor driver genes, we interrogated five data sources (see methods) to screen out known driver genes. Top mutated candidate driver genes documented in at least one of the data sources including BRWD1, FAT4, MYCBP2, NIN, ZNF292 (each 28.8%) etc. were annotated in Fig. 1A. APC was the candidate driver gene documented in all of the five data sources which had the highest mutation rates in the R-NET samples (4/18, 22.2%), followed by BRCA2 (3/18, 16.7%) (Supplementary Table 1). The total identified driver genes are provided in the Supplementary Table 1. The median TMB was 1.15 Muts/MB (range, 0.03–23.28) which was lower than the TMB of colorectum NEC (median: 5.18 Muts/MB) (Chen *et al.* 2020). TMB of three samples (16.7%) was more than 10. The median MSI was 0.36 (range, 0.00–10.97). Only one patient (5.6%) presented as MSI-H (MSI>10). For CNV, we found that regions of Chr 15q11.2 which covered the location of *GOLGA6L1*, *GOLGA6L22*, *GOLGA6L6*, *POTEB*, *POTEB2*, and *POTEB3* were amplified in a large proportion (83.3%) of patients, and regions of Chr 15q13.3 (covered location of GOLGA8K, GOLGA8N, and GOLGA8O), Chr 2q13 (covered *RGPD5*), and Chr 2q14.1 (covered *RGPD8*) were amplified in 77.8% of patients. Other common regions of amplification (13/18, 72.2%) included Chr 11q12.3 (covered *AHNAK*), Chr 15q11.1-q11.2 (covered *HERC2P3*,* OR4N4C*), Chr 1p36.21-p36.13 (covered *SPEN*), and etc. (Fig. 1B). CNV and copy number amplification (CNA) are most frequently found on Chr 19 and Chr 1 with incidence rates of 100% (18/18). Copy number loss (CNL) is most frequently observed on Chr 10 with an incidence rate of 61.1% (11/18) (Supplementary Fig. 1A). Besides, we detected no gene fusion events.

### Functional enrichment and signaling pathway analysis for frequently altered genes

**Figure 2:**
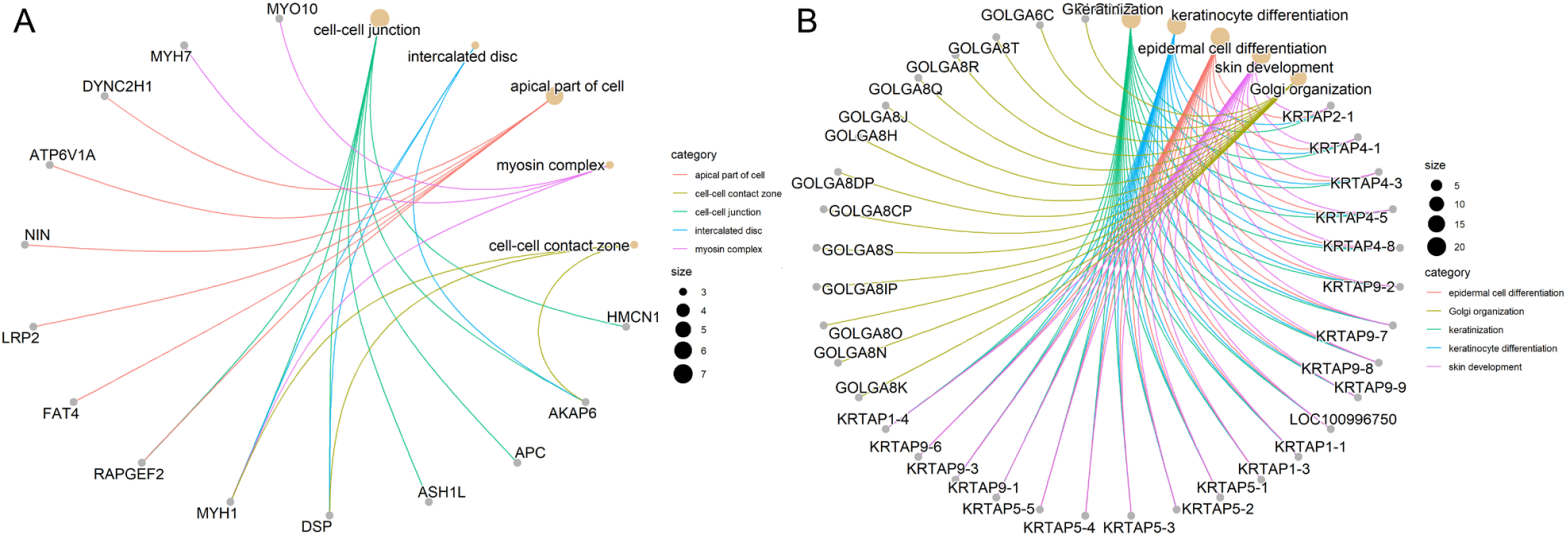
The enriched function of frequently altered genes. Enriched functions of top altered genes with mutation rates of more than 16.7% (A) and genes with CNV rates of more than 44.4% (B) were shown. Functional enrichment was based on Gene Ontology (GO). Enriched functions and the corresponding matched genes were linked with specific colored lines. A full-colour version of this figure can be found at https://doi.org/10.1530/ERC-22-0257.

**Figure 3:**
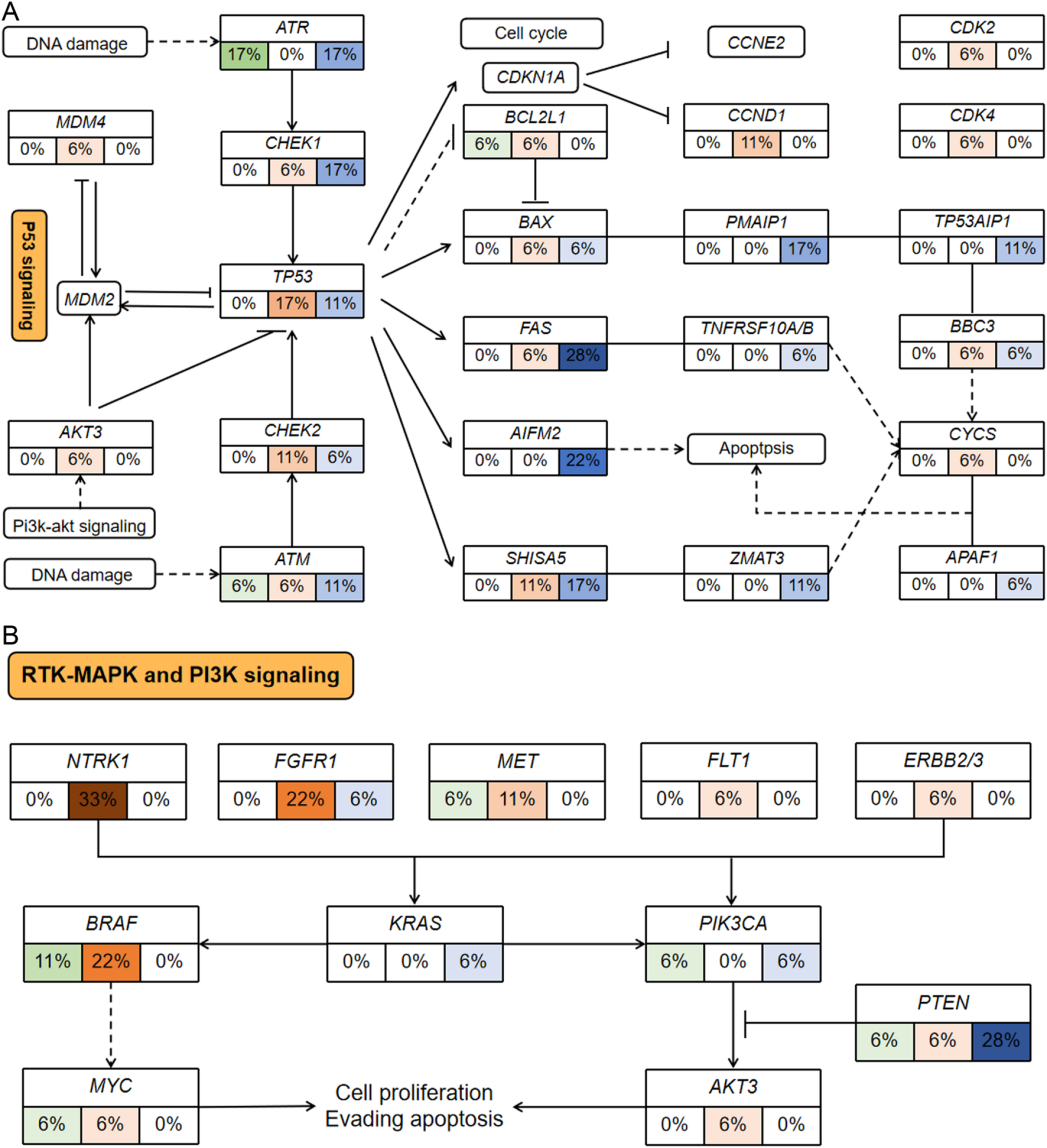
Part of highly altered signaling pathways in R-NETs. The frequency of somatic mutations and copy number variations is shown for key genes in the P53 signaling pathway (A), MAPK, and PI3K-AKT signaling pathway (B). Different colors represent different gene alterations. Green represents mutation, red represents copy number amplification, and blue represents copy number deletion. The darker the color, the higher the frequency. A full-colour version of this figure can be found at https://doi.org/10.1530/ERC-22-0257.

To investigate the biological function of frequently altered genes, we performed GO enrichment analysis for genes with mutational rates more than or equal to 16.7% (*n* = 169) and genes with CNV rates more than or equal to 44.4% (*n* = 183), respectively. Top enriched GO terms for frequently mutated genes included microtubule-based movement, regulation of cytokinesis, cell–cell junction, microtubule, the extrinsic component of membrane, ATPase activity, etc. (Supplementary Fig. 2A). Top enriched GO terms for genes with high-frequency CNV included keratinization, keratinocyte differentiation, epidermal cell differentiation, Golgi organization, endomembrane system organization, keratin filament, intermediate filament cytoskeleton, endopeptidase activity, etc. (Supplementary Fig. 2B). Enriched functions of top altered genes with mutation rates of more than 16.7% and genes with CNV of rates more than 44.4% and the corresponding matched genes were shown in Fig. 2. Next, we analyzed the gene alteration ratio and frequency of patients with gene alterations in each signaling pathway based on the Hallmark gene set in the Msigdb. We found all patients had alterations in the P53 pathway, mitotic spindle, estrogen response, E2F targets, and PI3K-AKT-mTOR signaling pathway (Supplementary Fig. 3A). Highly affected pathways in terms of altered gene ratio included mitotic spindle (with 75.9% of genes in this pathway altered), MYC targets v2 (altered gene ratio: 75.9%), reactive oxygen species (altered gene ratio: 75.5%), Notch signaling (altered gene ratio: 75.0%), apoptosis (altered gene ratio: 74.5%), etc. (Supplementary Fig. 3B). We further annotated the main altered genes with variation type and frequency in the P53 pathway, receptor tyrosine kinase (RTK)-MAPK, and PI3K signaling based on the signaling pathway maps in KEGG (Fig. 3). Other related frequently altered signaling pathways including WNT signaling, cell cycle, apoptosis, DNA repair (base excision repair, nucleotide excision repair, mismatch repair, homologous recombination repair, and non-homologous end-joining repair) were also mapped (Supplementary Fig. 4, 5, 6, 7, 8, 9, 10 and 11).

### Genetic alterations and survival

**Figure 4:**
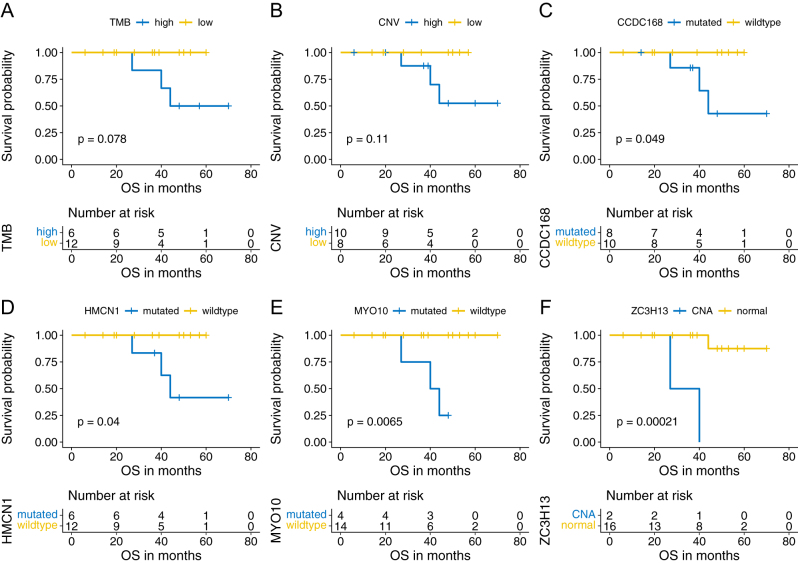
Genetic variations with significant correlation with overall survival (OS). Survival analysis indicated that patients with higher TMB (*P* = 0.078) (A), higher CNV (*P* = 0.110) (B) tended to have worse OS. Patients with mutation of *CCDC168* (*P* = 0.049) (C), *HMCN1* (*P* = 0.040) (D), *MYO10* (*P* = 0.007) (E), and copy number amplification (CNA) of* ZC3H13* (*P* < 0.001) (F) had significantly worse OS in R-NETs. A full-colour version of this figure can be found at https://doi.org/10.1530/ERC-22-0257.

To explore the potential correlation of gene alterations with OS, we followed up on these 18 patients. In the whole cohort, the median follow-up time was 39.5 months. Three patients (3/18, 16.7%) died at the last follow-up. According to the optimal cut-off value of TMB, CNV, and MSI (TMB: 3.08/MB; CNV: 4.30E+7, MSI: 0.15) derived from the maximally selected rank statistics by the R package survminer, patients were divided into two groups respectively. Patients with higher TMB or CNV tended to have shorter OS (TMB: *P* = 0.078; CNV: *P* = 0.11, Fig. 4A and B), while MSI did not have a prognostic significance (MSI: *P* = 0.26). For somatic mutation, we found significant correlation of *CCDC168*, *HMCN1, MYO10* mutation with worse OS (*CCDC168*: *P* = 0.049; *HMCN1*: *P* = 0.040; *MYO10*: *P* = 0.007, Fig. 4C, D, and E). For CNV, we found a significant correlation of *ZC3H13* CNA with worse OS (*P* < 0.001, Fig. 4F). We also investigated the correlation of gene variation involved in the P53 signaling pathway and PI3K-AKT-pathway with prognosis. We found patients with mutation of *ITGB8* (*P* = 0.032), *BRCA1* (*P* = 0.014), *SGK1* (*P* = 0.014) and CNL of *APAF1* (*P* < 0.001) had significantly shorter OS (Supplementary Fig. 12A, B, C and D).

### Pathway alterations and survival

**Figure 5:**
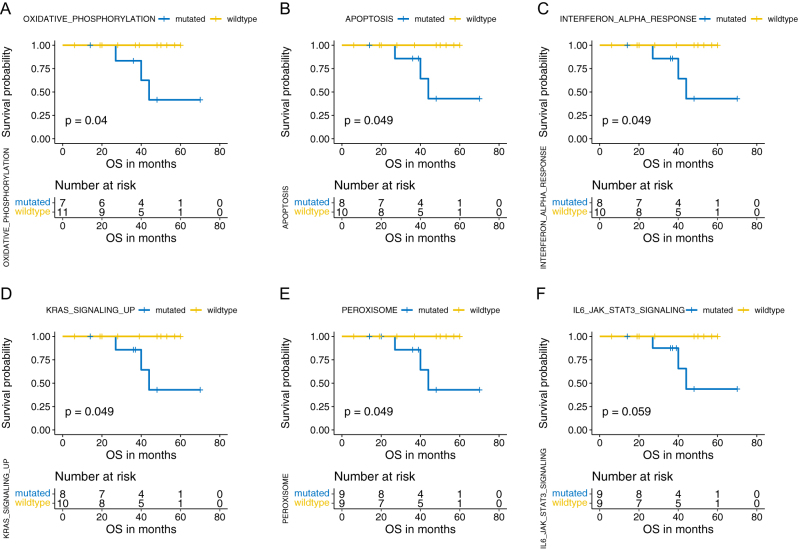
Signaling pathway alteration with significant correlation with overall survival (OS). Survival analysis indicated that gene mutations in pathways of oxidative phosphorylation (*P* = 0.040) (A), apoptosis (*P* = 0.049) (B), interferon alpha response (*P* = 0.049) (C), KRAS signaling up (*P* = 0.049) (D), and peroxisome (*P* = 0.049) were significantly correlated with worse OS in R-NETs. Gene mutations in the IL6-JAK-STAT3 signaling pathway tended to associate with worse OS with a *P* value of 0.059. Included signaling pathways and corresponding gene sets were derived from Hallmark gene sets in the Molecular Signatures Database (Msigdb). A full-colour version of this figure can be found at https://doi.org/10.1530/ERC-22-0257.

The correlation of pathway alterations with survival was investigated based on the Hallmark gene set. In total, we found five pathways with gene mutations significantly correlated with worse OS: oxidative phosphorylation (*P* = 0.040), apoptosis (*P* = 0.049), interferon alpha response (*P* = 0.049), KRAS signaling up (*P* = 0.049), and peroxisome (*P* = 0.049) (Fig. 5A, B, C, D and E). Besides, patients with gene mutations in L6-JAK-STAT3 signaling tended to have shorter OS (*P* = 0.059) (Fig. 5F).

### Potentially targetable gene alterations for targeted therapy

**Figure 6:**
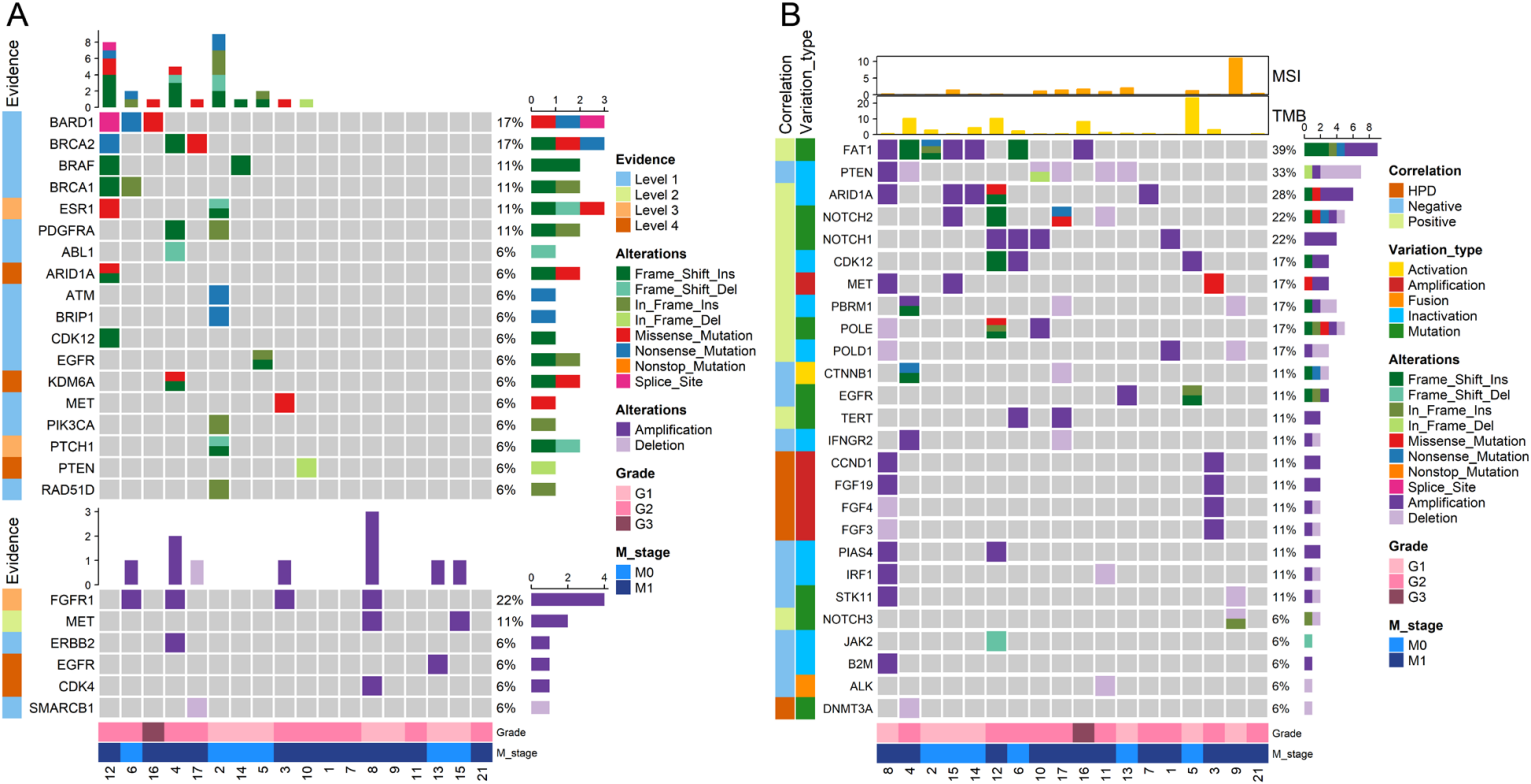
Gene alterations associated with therapy. Potentially actionable gene alterations for targeted therapy (A) and potential gene alterations with impact on the efficacy of immune checkpoint blockade (ICB) (B). Gene lists for targeted therapy and ICB were selected based on the Oncology KnowledgeBase (oncoKB) database and literature reports respectively. Clinicopathologic and genetic parameters were annotated in the top bar plots and bottom colored bars. A full-colour version of this figure can be found at https://doi.org/10.1530/ERC-22-0257.

To find potentially targetable gene alterations (PTGAs) for R-NETs, the frequency of gene alterations annotated by OncoKB was counted in our cohort. In total, 13/18 (72.2%) of the patients had at least one gene alteration for all-level targets documented in the OncoKB database, which included 10/18 (55.6%) of patients who harbored at least one targetable gene mutation and 7/18 (38.9%) of patients who harbored at least one targetable gene CNV. Of these PTGAs, targetable gene mutations with top frequency included *BARD1*, *BRCA2* (each *n* = 3, 16.7%), *BRAF*, *BRCA1*, *ESR1*, and *PDGFRA* (each *n* = 2, 11.1%) (Fig. 6A). Besides, mutation of *ABL1*, *ARID1A*, *ATM*, *BRIP1*, *CDK12*, *EGFR*, *KDM6A*, *MET*, *PIK3CA*, *PTCH1*, *PTEN*, and *RAD51D* was each found in one patient (each *n* = 1, 5.5%). For potentially targetable CNVs, *FGFR1* amplification was the most prevalent CNV with a frequency of 22.2% (*n* = 4), followed by *MET* amplification (*n* = 2, 11.1%). Other potentially targetable CNVs included amplification of *CDK4*, *EGFR*,* ERBB2*, and deletion of* SMARCB1* (each *n* = 1, 5.5%) (Fig. 6A). The total targetable gene list and corresponding drugs were shown in Supplementary Table 2.

### Gene alterations potentially influencing immune checkpoint blockade

In addition to targeted therapy, we also evaluated patients carrying gene alterations associated with the efficacy of ICB. Among 33 genes reported to be correlated with response to ICB, 26 genes (78.8%) had variation in our cohort (the total gene list was shown in Supplementary Table 3). We found that 17/18 (94.4%) of the patients carried these gene alterations potentially influencing the efficacy of ICB (Fig. 6B). *FAT1* gene alterations were the most common gene alterations potentially influencing the activity of ICB (*n* = 7, 38.9%, including three mutations and four amplifications, Fig. 6B). In total, the negative genetic predictors of ICB were found in eight patients (44.4%). *PTEN* inactivation was the most common gene alteration found to negatively influence the response to ICB (*n* = 5, 27.8%, including one patient with concurrent *PTEN* mutation and deletion and four patients with *PTEN* deletion, Fig. 6B). Besides, mutation of *CTNNB1*, *EGFR*, and *JAK2* and deletion of *IFNGR2*, *IRF1*, and *STK11* each were found in one patient (5.5%). In addition, gene alterations associated with hyperprogression (HPD) included amplification of *CCND1* (*n* = 2, 11.1%), *FGF3* (*n* = 1, 5.5%), *FGF4* (*n* = 1, 5.5%), and *FGF19* (*n* = 2, 11.1%) and deletion of *DNMT3A* (*n* = 1, 5.5%) were found in total three patients (16.7%).

## Discussion

To our knowledge, this study is the first to exclusively depict the unique genomic characteristics of R-NETs using whole-exome sequencing in the Chinese population. We found the top mutated genes were significantly distinct from the results using the targeted gene panel. More genetic variations were detected by whole-exome sequencing. Genes like *CCDC168*, *PPP4R3A*, *GALNT11*, and *HMCN1* were frequently mutated which have not been reported in previous studies in R-NENs (Chen *et al.* 2020, 2021). Functional enrichment analysis showed these frequently altered genes are mainly involved in microtubule-based movement, regulation of cytokinesis, cell–cell junction, microtubule, the extrinsic component of membrane, ATPase activity, etc. Besides, a large number of high-frequency CNV events were found, like the amplification of *GOLGA6L6*, *POTEB3*, *POTEB2*, *POTEB*, *LOC102723623*, *GOLGA6L1*, and *GOLGA6L22*. These genes mainly function in keratinization, keratinocyte differentiation, epidermal cell differentiation, Golgi organization, endomembrane system organization, keratin filament, intermediate filament cytoskeleton, endopeptidase activity, etc. However, further functional studies are needed to validate the biological effects of these genes in R-NETs. Signature 1A and G: C>A: T were the most prevalent mutation characteristic in this cohort. Signature 1A was related to the elevated rate of spontaneous deamination of 5-methyl-cytosine which results in C>T transitions and showed a strong association with age (Alexandrov *et al.* 2013). The predominant of signature 1A might reflect that the etiology of R-NET was mainly related to age but lack of carcinogen exposures. Besides, signature 3 is found in half of the patients which is associated with *BRCA1* and *BRCA2* mutation, implicating that the deficiency of homologous recombination repair might contribute to the pathogenesis of R-NET. Previous studies investigating the CNVs of NEN were mainly limited to the gastrointestinal tract and pancreas (Puccini *et al.* 2020). Our study might provide unique insight for understanding the genetic characteristics of NET in the rectum.

Previous genomic studies of R-NENs were mainly performed in CR-NECs, with frequently mutated genes including *TP53* (24–65.5%)*, RB1* (4–16.7%)*, APC* (16–59.5%)*, KRAS* (24–36.9%), *BRAF* (8.3–20.2%), etc. (Chen *et al.* 2020, 2021, Venizelos *et al.* 2021). In general, these gene alterations were scarcely observed in our R-NETs cohort, except for *APC*, which had a mutation rate of 22.2% (4/18) in our R-NET cohort (Supplementary Fig. 13). *APC* was also frequently mutated in COREAD (55–96%) (Conlin *et al.* 2005, Chen *et al.* 2020). However, previous studies had found *APC* had a lower frequency of mutation in pancreatic NENs (1–15%) (Puccini *et al.* 2020, Venizelos *et al.* 2021), esophagus NEC (11%) (Venizelos *et al.* 2021), and gastric NEC (6%) (Venizelos *et al.* 2021). This indicated that *APC* mutation was tumor-site specific but not dependent of tumor grade and differentiation. For *TP53*, we found no mutation but two deletions. For *RB1*, one patient had the insertional mutation and the other one patient had *RB1* deletion. All patients with *TP53* and/or *RB1* alterations were in low grade in this study. This suggests that *TP53* and *RB1* inactivation can occur in low-grade NETs but had a lower frequency compared with NECs. We found no *KRAS* mutations, indicating *KRAS* is likely to mainly function in the CR-NECs instead of R-NETs. However, we found four insertional mutations of *BRAF* in two R-NET patients (11.1%) and four additional *BRAF* amplifications (22.2%). Besides, three *BRCA* mutations (16.7%), five *PTEN* deletions, and one *PTEN* mutation (27.8%) were found in our cohort which might be the characteristic molecular alteration in R-NET (Supplementary Fig. 13).

In this study, we found that all patients had at least one gene alteration in the P53 signaling pathway, indicating the pivotal role of the P53 signaling pathway in R-NETs. The P53 signaling pathway was involved in the regulation of DNA damage repair, cell cycle, and apoptosis. We found that key genes promoting apoptosis, such as *FAS* (28%), *PRF1* (28%), *CASP7* (22%), *AIFM2* (22%), *TNFSF10* (22%), and *MAPK8* (22%) had high-frequency copy number deletion (CND), while *NTRK1* (33%), *FGFR1* (22%), *MET* (11%), *BRAF* (22%) in the RTK-MAPK pathway which promoted proliferation had high-frequency CNA. In addition, *PTEN* (28%), the key negative regulator of the PI3K-AKT-mTOR pathway had frequent depletion. These alterations might lead to abnormal activation of the downstream cell proliferation signal. We speculated that the frequent variation in MAPK and PI3K-AKT signaling pathway might contribute to the relatively high sensitivity of R-NETs to the mTOR inhibitor Everolimus. In addition, we found that the DNA damage repair pathway also had frequent alterations. The DNA damage sensors, *ATM* (mutation: 6%, deletion: 11%) and *ATR* (mutation: 17%, deletion: 17%), were frequently mutated and depleted. Among various DNA damage repair mechanisms, genes involved in homologous recombination and nucleotide excision repair had relatively higher mutation rates, such as *BRCA1* (11%), *BRCA2* (17%), *BARD1* (17%), and *ERCC6* (17%). While genes involved in non-homologous end-joining repair had relatively higher rates of CNL, such as *POLL* (22%) and *DNTT* (28%). We found that the key genes involved in mismatch repair, such as *MLH1*, *MSH2*, and *MSH3*, mainly had CNV but rarely mutated. In addition, genes like *APC* (33%), *WNT5B* (11%), and *AXIN2* (11%) in the WNT signaling pathway that regulates cell proliferation and differentiation frequently exhibited CNA. Simultaneously, *BIRC5* (11%), *MYC* (6%), and *CCND1* (11%), the downstream molecules of WNT signaling, also showed CNA, suggesting activation of the WNT-β catenin signaling pathway in a subset of patients.

We found higher TMB was associated with poor prognosis which was consistent with the previous report (Chen *et al.* 2020). For single gene variation, we found a mutation of* CCDC168*, *HMCN1*, *MYO10*, and CNA of *ZC3H13* significantly correlated with worse OS. *CCDC168* is a protein-coding gene with a poorly understood function known to be mutated in several cancer types (Wang *et al.* 2018). *CCDC168* is reported to have a frequent mutation in pediatric renal cell carcinomas (Beck *et al.* 2022), malignant pleural mesothelioma (Yu *et al.* 2021), and colon adenocarcinoma (Cortes-Ciriano *et al.* 2017). Our study identified *CCDC168* as the top mutant gene and associated with poor OS in R-NETs. *HMCN1* encodes a large extracellular member of the immunoglobulin superfamily. *HMCN1* is frequently mutated in prostate cancer (Zhao *et al.* 2019) and gastric and colorectal cancer (Lee *et al.* 2015). Mutation of *HMCN1* is associated with poor prognosis in clear cell renal carcinoma (Gong *et al.* 2022) and breast cancer (Kikutake *et al.* 2018). Besides, it was found to be associated with higher TMB in ovarian cancer (Lu *et al.* 2021). In our study, we also found R-NETs with *HMCN1* mutation exhibited higher TMB (median: 6.39 vs 0.60, *P* = 0.010). The underlying mechanism of *HMCN1* mutation to potentially cause high TMB and the potential link between *HMCN1* mutation and response to ICB are interesting to be further studied. *MYO10* encodes a member of the myosin superfamily which functions as an actin-based molecular motor and plays a role in the integration of F-actin and microtubule cytoskeletons during meiosis. *MYO10* is found to be upregulated in many humor cancers and correlated with poor prognosis, such as lung squamous cell carcinoma (Dvornikov *et al.* 2018) and cervical cancer (He *et al.* 2020). High expression of *MYO10* contributes to the tumor invasion and migration of breast cancer (Cao *et al.* 2014), prostate cancer (Makowska *et al.* 2015), and colorectal cancer (Ou *et al.* 2022). Our study first reported that *MYO10* mutation is associated with poor prognosis in R-NETs. Evidence has shown *MYO10* promotes tumor progression by inducing genomic instability which in turn creates an immunogenic environment for ICB (Mayca Pozo *et al.* 2021). In this study, we found patients with *MYO10* mutations had higher TMB (median: 6.39 vs 0.65 Muts/MB, *P* = 0.029), supporting the role of *MYO10* in the regulation of genomic stability. However, the correlation of *MYO10* mutation with response to ICB remains to be further elucidated in R-NETs. *ZC3H13* was a part of the RNA N6-methyladenosine methyltransferase complex involved in RNA epigenetic modification, which was reported to be a tumor suppressor in colorectal cancer, breast cancer, and endometrial cancer (Zhu *et al.* 2019, Gong *et al.* 2020, Ma *et al.* 2021). However, in our study, it seemed to be an unfavorable molecule since patients with *ZC3H13* CNA had worse OS. These gene alterations might play important roles in the development and progression of R-NETs and might be potential therapeutic targets that deserved to be further investigated.

Our results showed that up to 13/18 (72.2%) of the R-NETs had at least one PTGA, suggesting the promising prospect of targeted therapy in R-NETs. Our study showed up to 22% of the R-NETs harbored *FGFR1* amplification which was the most common PTGA, followed by *BARD1* mutation and* BRCA2* mutation (each 17%). Drugs targeting the FGFR1 amplification included infigratinib, Debio1347, and AZD4547, all of which were FGFR1-3 inhibitors. Infigratinib and AZD4547 had shown activity in lung squamous cell carcinoma with *FGFR1* amplification (Zhang *et al.* 2012, Nogova *et al.* 2017, Paik *et al.* 2017). Debio 1347 had also demonstrated encouraging efficacy across multiple solid tumor types with *FGFR1* amplification (Voss *et al.* 2019). However, these drugs had not been attempted in neuroendocrine tumors. Further preclinical studies and clinical trials are needed to test the efficacy of the *FGFR* inhibitors in R-NETs. Besides, our study suggested the wide range of adaptability of olaparib in R-NETs since 33.3% (6/18) of the patients harbored the targetable alterations for olaparib (including mutation of *BARD1*, *BRCA2* (each 16.7%), *BRCA1* (11.1%), *ATM*, *BRIP1*, *CDK12*, and *RAD51D* (each 5.6%)). However, olaparib had not been used in R-NETs. Besides, we analyzed all the variation types of potentially targetable genes validated in other malignancies. We found *NTRK1* amplification in six (33.3%) patients. *ROS1* was mutated in two patients and was amplified in four additional patients. However, drugs targeting these specific variation types mostly have not been developed yet. It remained to see whether the currently approved drugs targeting *NTRK1* rearrangements and *ROS1* fusions, entrectinib also had effectiveness in R-NETs. *BRAF* was not only mutated in two patients but also amplified in an additional four patients. However, currently approved drugs only target BRAF V600 mutations like dabrafenib and vemurafenib. The efficacy of these drugs for patients with *BRAF* amplification is unsure and specific drugs targeting the *BRAF* amplification are needed. In sum, these frequently altered genes are mostly involved in RTK-MAPK and PI3K-AKT signaling indicating that targeting these signaling pathways might be promising therapeutic strategies for R-NETs. This was supported by the exceptional efficacy of the mTOR inhibitor, Everolimus, implicated in advanced G1-2 R-NENs (Yao *et al.* 2016). Moreover, Surufatinib, the novel approved multitargeted receptor tyrosine kinase inhibitor (RTKI) targeting *VEGFR1-3*, *FGFR1*, and *CSF1R* has also demonstrated benefit for patients with advanced extra-pancreatic NETs in China (Xu *et al.* 2020). Other novel RTKIs like cabozantinib, lenvatinib, and pazopanib have been developed and shown promising efficacy (Das & Dasari 2021). With a relatively higher ORR of 15% for extra-pancreatic NETs and well tolerance observed in the phase II study (Chan *et al.* 2014), the randomized phase III CABINET trial of cabozantinib for advanced extra-pancreatic NETs has been initiated (NCT03375320), which may bring better efficacy and safety in R-NET patients.

ICB represented a promising strategy for cancer treatment in a broad range of malignancies. However, the immune checkpoint inhibitors (ICIs) targeting PD-(L)1 and/or CTLA-4 have not seen clinical meaningful anti-tumor effects in gastroenteropancreatic (GEP) -NETs, except for the rare subsets of patients with MSI-H or TMB-H (Mehnert *et al.* 2020, Patel *et al.* 2020, Chan *et al.* 2021). In our study, the overall TMB and MSI were extremely low with only three patients having TMB-H and one patient having MSI-H, which was concordant with the previous reports (Mitsuhashi *et al.* 2015, Chen *et al.* 2020, Venizelos *et al.* 2021). The overall low TMB and MSI in GEP-NETs might contribute to the overall poor response to immunotherapy. Nevertheless, ICB might serve as an alternative option for selected patients with TMB-H given that patients with higher TMB suffered from worse OS. Besides the widely accepted predictive biomarkers, TMB and MSI, we investigated other genetic predictors of response to ICB reported in previous literature. We found 44.4% of the patients harbored at least one negative genetic predictor of ICB. *PTEN* depletion (*n* = 5, 27.8%) was the most common gene alteration found to potentially negatively influence the response to ICB. *PTEN* depletion was found to be correlated with immune resistance and tumor immune evasion and predicted poor response to ICB in a variety of malignancies (Peng *et al.* 2016, Vidotto *et al.* 2020, Lin *et al.* 2021). Besides, *FAT1* was also frequently altered with four patients harboring CNAs and three patients harboring insertional mutations. Previous studies had indicated a positive correlation of *FAT1* mutation with the durable clinical benefit of ICB in non-small cell lung cancer (Fang *et al.* 2019, Feng *et al.* 2022). However, conflicting results were shown in another data-mining study (Zhu *et al.* 2021). Our study showed that *FAT1* was not only mutated but also had relatively high-frequency CNA in R-NETs. Further studies are needed to validate the potential effects of *FAT1* multi-type variations on response to ICB. Other genetic negative predictors found in our cohort but with a low frequency included mutation of *CTNNB1*, *EGFR*, and *JAK2* and deletion of *IFNGR2*, *IRF1*, and *STK11* (each *n* = 1). Notably, gene alterations associated with HPD were found in a total of three patients (16.7%), which underscores the need for real-time imaging monitoring for R-NET with these alterations when receiving ICB and the value of genetic testing upfront of the therapy. Oncogenic pathway activation was found as a critical tumor-intrinsic mechanism for resistance to ICB (Kalbasi & Ribas 2020). Given frequent gene alterations in the MAPK, PI3K-AKT, and WNT signaling pathway observed in this study, further combinational strategies concurrently targeting these pathways and negative immune checkpoints are deserved to be tested in R-NETs.

There were some limitations in this work. First, this study was performed in a single center and the retrospective nature may lead to some bias. Secondly, the sample size was limited. However, given the rarity of R-NETs, including a large number of patients was difficult. Thirdly, two samples were collected after treatment which might influence the sequencing results. Fourthly, sample types are heterogeneous and this might further impact the results to a certain degree. However, given the complexity of the realistic situations, the specimen types can be diverse. Since only 3 fresh tumor tissues were included, and all of the remaining 15 samples are FFPE tissues. We believed that this might have a much smaller impact on the heterogeneity of the results than the inherent heterogeneity between tumor tissues. Further genomic studies with larger sample sizes and samples of homogeneity are needed.

In summary, multiple oncogenic pathways were frequently altered potentially leading to the development and progression of R-NETs. Several gene alterations were found to be significantly associated with worse OS which might be potential therapeutic targets. Targeted therapy might be a promising strategy as targetable gene alterations were prevalent in a high fraction of R-NETs. *FAT1* alteration and *PTEN* depletion might be the main genetic alterations influencing the response to ICB besides overall low TMB and MSI in R-NETs.

## Supplementary Materials

Table S1

Table S2

Table S3 Gene alterations with potential correlation with the efficacy of immune checkpoint blockade.

Figure S1. Characteristic of somatic mutation in rectal neuroendocrine tumors (R-NETs). The distribution of frequency of patients with altered genes on 24 chromosomes (a). The upper panel showed the frequency of patients with gene mutations and the lower panel showed the frequency of patients with gene copy number variations (CNVs). CNG: copy number gain. CNL: copy number loss. Clustering of R-NETs based on the mutational spectrum of included patients (b). Clustering of R-NETs based on the mutational signature of included patients (c).

Figure S2. Functional enrichment analysis for frequently mutated genes (a) and genes with high-frequency copy number variations (CNVs) based on the Gene Ontology database. BP: biological process; CC: cellular components; MF: molecular function.

Figure S3. The bar plots show the frequency of patients with gene alterations (a), and the altered gene ratio (b) in 50 Hallmark signaling pathways.

Figure S4. The frequency of somatic mutations and copy number variations are shown for key genes in the WNT canonical signaling pathway based on KEGG. Green represents mutation, red represents copy number amplification, and blue represents copy number deletion. The darker the color, the higher the frequency.

Figure S5. The frequency of somatic mutations and copy number variations are shown for key genes in the Cell cycle signaling pathway based on KEGG. Green represents mutation, red represents copy number amplification, and blue represents copy number deletion. The darker the color, the higher the frequency.

Figure S6. The frequency of somatic mutations and copy number variations are shown for key genes in the Apoptosis signaling pathway based on KEGG. Green represents mutation, red represents copy number amplification, and blue represents copy number deletion. The darker the color, the higher the frequency.

Figure S7. The frequency of somatic mutations and copy number variations are shown for key genes in the Base excision repair signaling pathway based on KEGG. Green represents mutation, red represents copy number amplification, and blue represents copy number deletion. The darker the color, the higher the frequency.

Figure S8. The frequency of somatic mutations and copy number variations are shown for key genes in the Nucleotide excision repair signaling pathway based on KEGG. Green represents mutation, red represents copy number amplification, and blue represents copy number deletion. The darker the color, the higher the frequency.

Figure S9. The frequency of somatic mutations and copy number variations are shown for key genes in the Mismatch repair signaling pathway based on KEGG. Green represents mutation, red represents copy number amplification, and blue represents copy number deletion. The darker the color, the higher the frequency.

Figure S10. The frequency of somatic mutations and copy number variations are shown for key genes in the Homologous recombination repair signaling pathway based on KEGG. Green represents mutation, red represents copy number amplification, and blue represents copy number deletion. The darker the color, the higher the frequency.

Figure S11. The frequency of somatic mutations and copy number variations are shown for key genes in the Non-homologous end-joining repair signaling pathway based on KEGG. Green represents mutation, red represents copy number amplification, and blue represents copy number deletion. The darker the color, the higher the frequency.

Figure S12. Gene alterations with significant correlation with overall survival (OS) in the PI3K-AKT pathway and the P53 signaling pathway. Survival analysis indicated that patients with mutation of ITGB8 (P = 0.032) (a), BRCA1 (P = 0.014) (b), SGK1 (P = 0.014) and copy number loss (CNL) of APAF1 (P < 0.001) (d) had significantly worse OS in R-NETs.

Figure S13. The variation frequency and types of common gene variations of neuroendocrine neoplasms (NENs) were reported in previous genomic studies in our rectal NET cohort.

## Declaration of interest

The authors declare that there is no conflict of interest.

## Funding

This work was supported by the National Key Research and Development Program of Chinahttp://dx.doi.org/10.13039/501100012166 (grant number 2019YFB1309704).

## Data availability

Sequencing data are available at the request of the corresponding authors.

## Author contribution statement

LYL, CZX, TC, CYY, and SF collected the patients and samples; CZX, YFH, and CRA collected the clinicopathological information; SYF and LJ performed pathological review, GYY and ZSH performed the bioinformatic data analysis; LYL, GYY, and WZZ drafted the manuscript; THY and LJ directed the project and revised the manuscript.
