# Supplementary material for: Whole-exome sequencing of rectal neuroendocrine tumors
Source: Endocr Relat Cancer. 2023 Aug 2;30(9):e220257. doi: 10.1530/ERC-22-0257 (PMC10450454; doi:10.1530/ERC-22-0257)
Supplement: Table S3 Gene alterations with potential correlation with the efficacy of immune checkpoint blockade. [file supplementary_table_3.pdf]

Table S3 Gene alterations with potential correlation with the efficacy of immune checkpoint blockade.

Gene	Alteration	Correlation
ARID1A	Inactivation	Positive
CD274	Amplification	Positive
CDK12	Inactivation	Positive
PBRM1	Inactivation	Positive
MET	Amplification	Positive
POLD1	Inactivation	Positive
POLE	Mutation	Positive
FAT1	Mutation	Positive
LRP1B	Mutation	Positive
NOTCH1	Mutation	Positive
NOTCH2	Mutation	Positive
NOTCH3	Mutation	Positive
TERT	Mutation	Positive
B2M	Inactivation	Negative
CTNNB1	Activation	Negative
JAK1	Inactivation	Negative
JAK2	Inactivation	Negative
EGFR	Mutation	Negative
ALK	Fusion	Negative
KEAP1	Mutation	Negative
PTEN	Inactivation	Negative
STK11	Mutation	Negative
SOCS1	Amplification	Negative
IFNGR1	Inactivation	Negative
IFNGR2	Inactivation	Negative
IRF1	Inactivation	Negative
PIAS4	Inactivation	Negative
CCND1	Amplification	HPD
FGF3	Amplification	HPD
FGF4	Amplification	HPD
FGF19	Amplification	HPD
MDM2	Amplification	HPD
DNMT3A	Mutation	HPD

Abbreviation: HPD: hyperprogression disease.
