# Supplementary figures and images for: Whole-exome sequencing of rectal neuroendocrine tumors
Source: Endocr Relat Cancer. 2023 Aug 2;30(9):e220257. doi: 10.1530/ERC-22-0257 (PMC10450454; doi:10.1530/ERC-22-0257)

**a**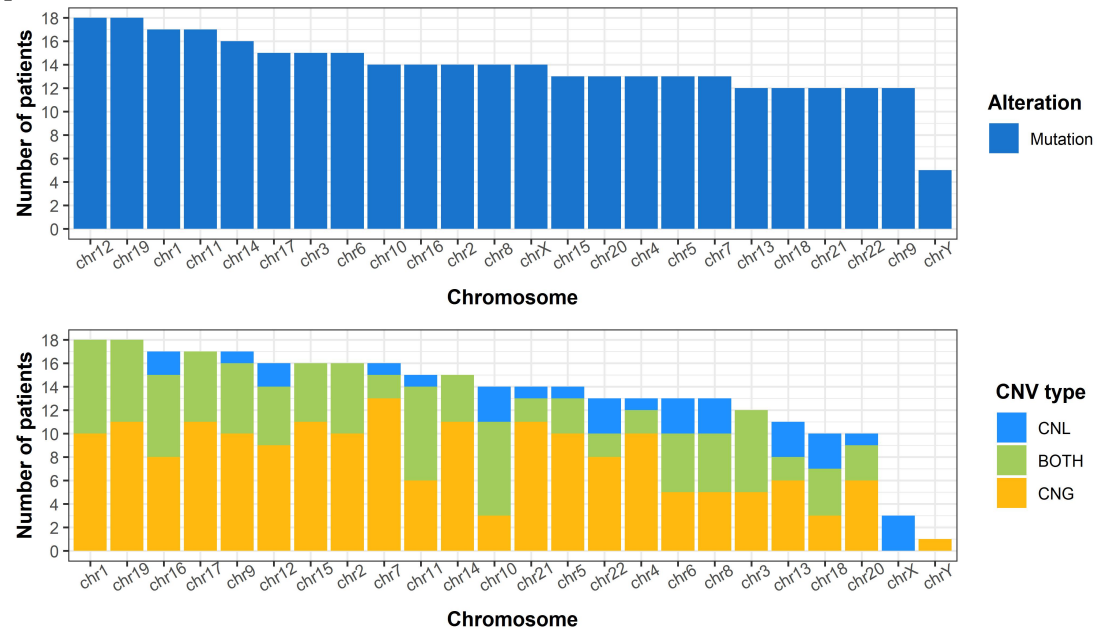**b**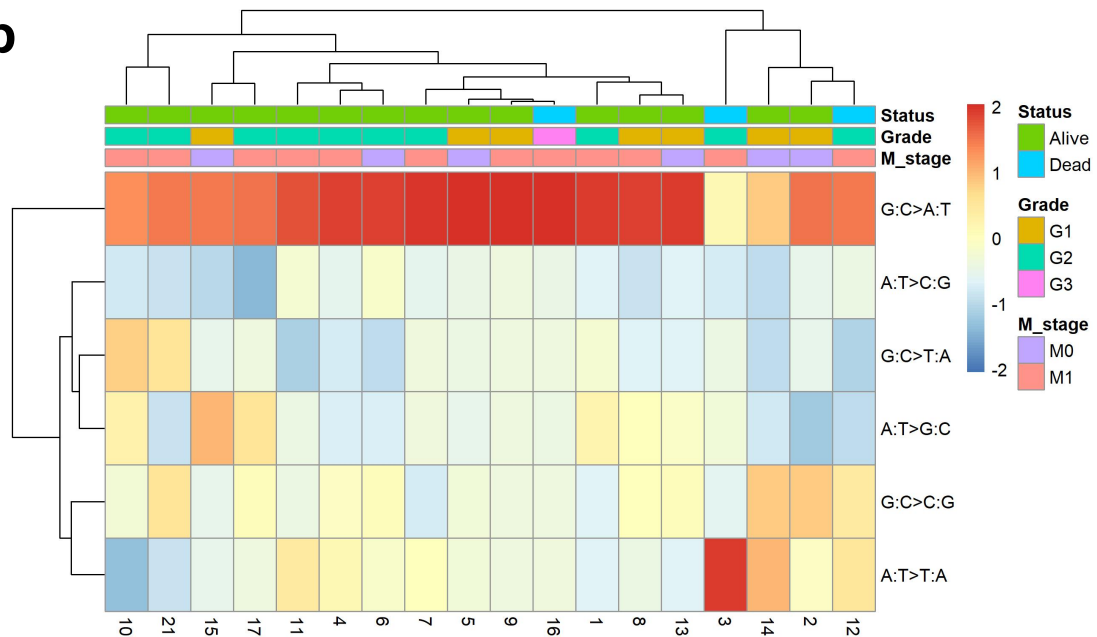**c**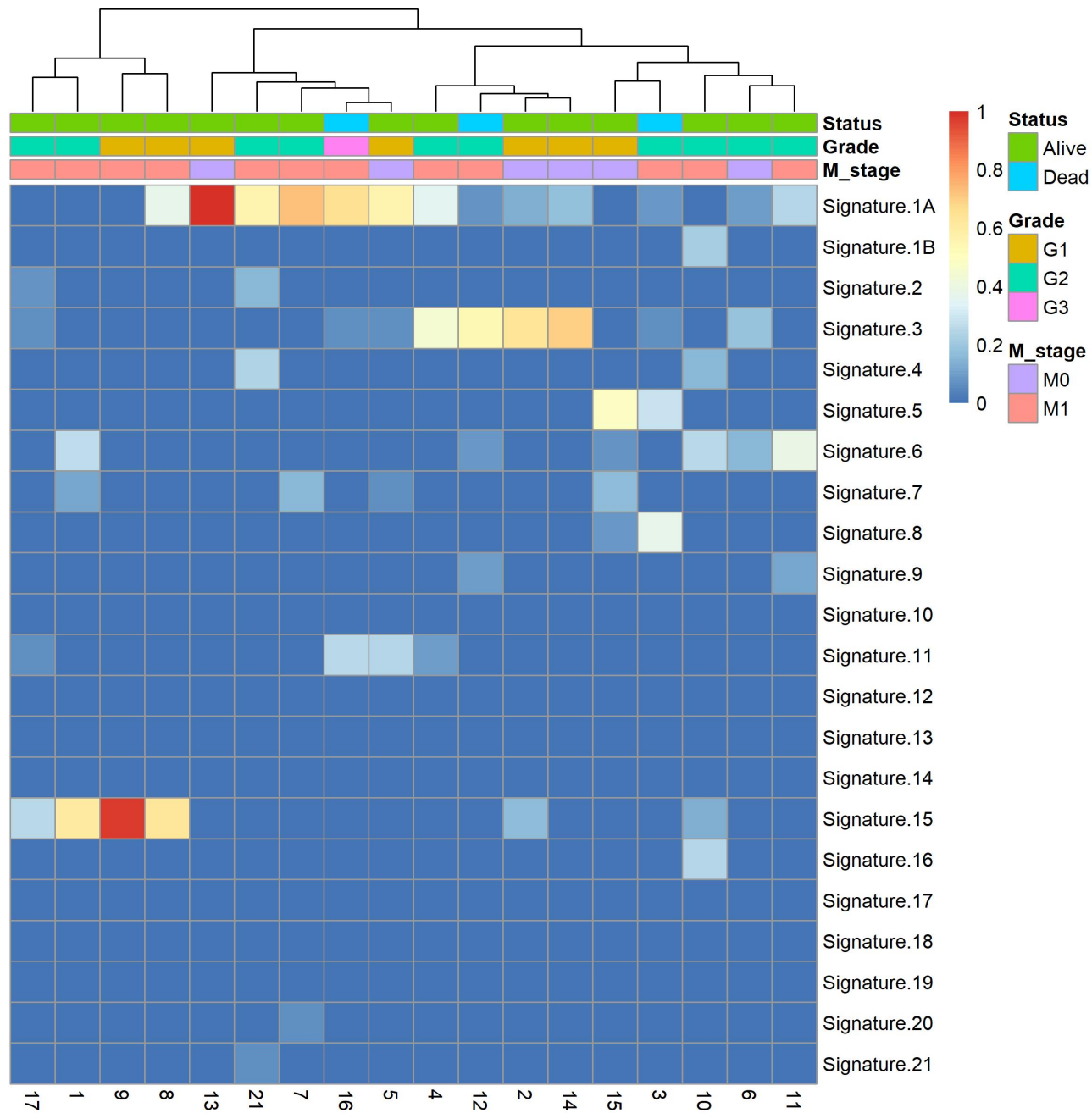

Supplement: Figure S1. Characteristic of somatic mutation in rectal neuroendocrine tumors (R-NETs). The distribution of frequency of patients with altered genes on 24 chromosomes (a). The upper panel showed the frequency of patients with gene mutations and the lower panel showed the frequency of patients with g [file supplementary_figure_1.pdf]

**a**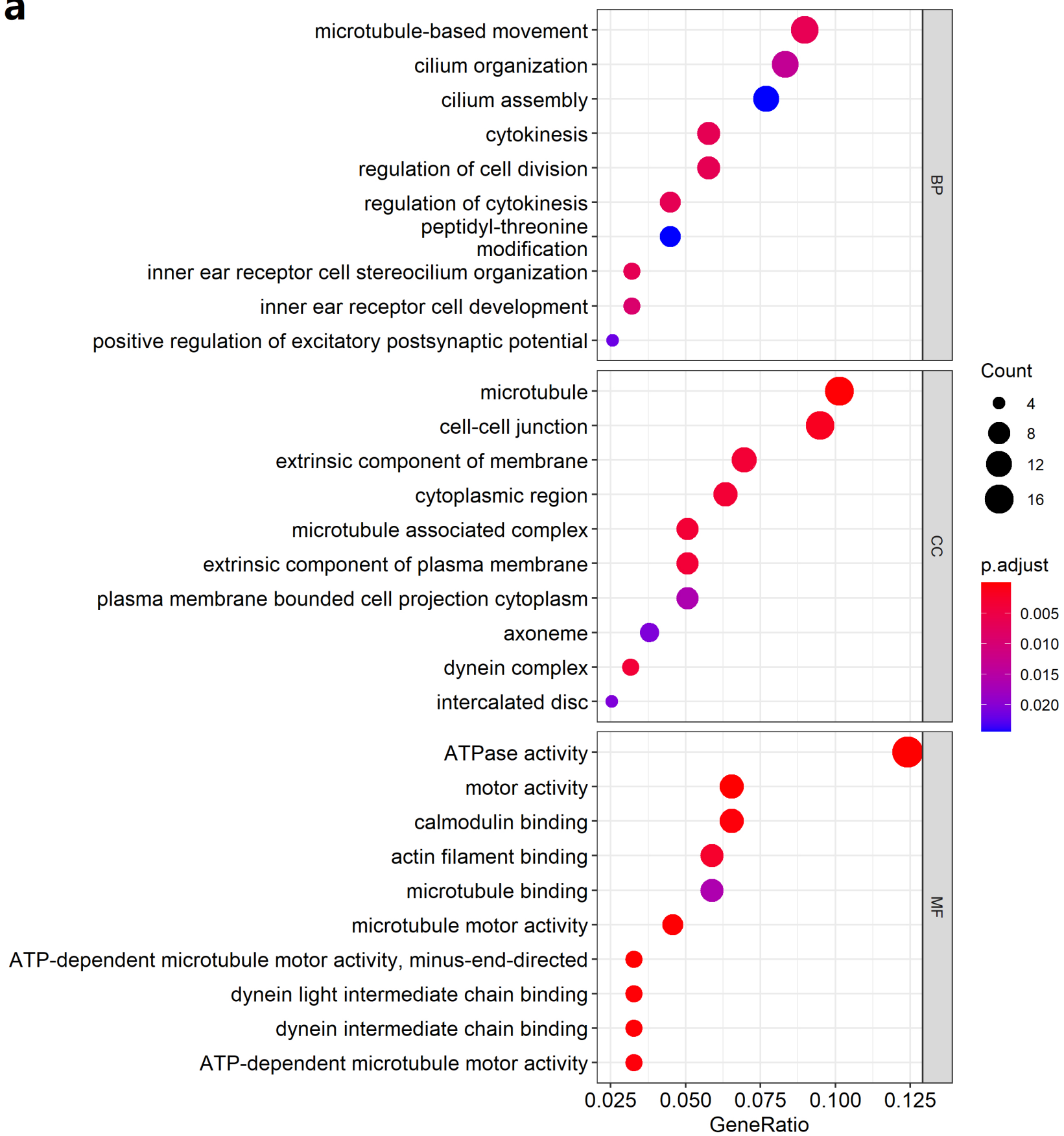**b**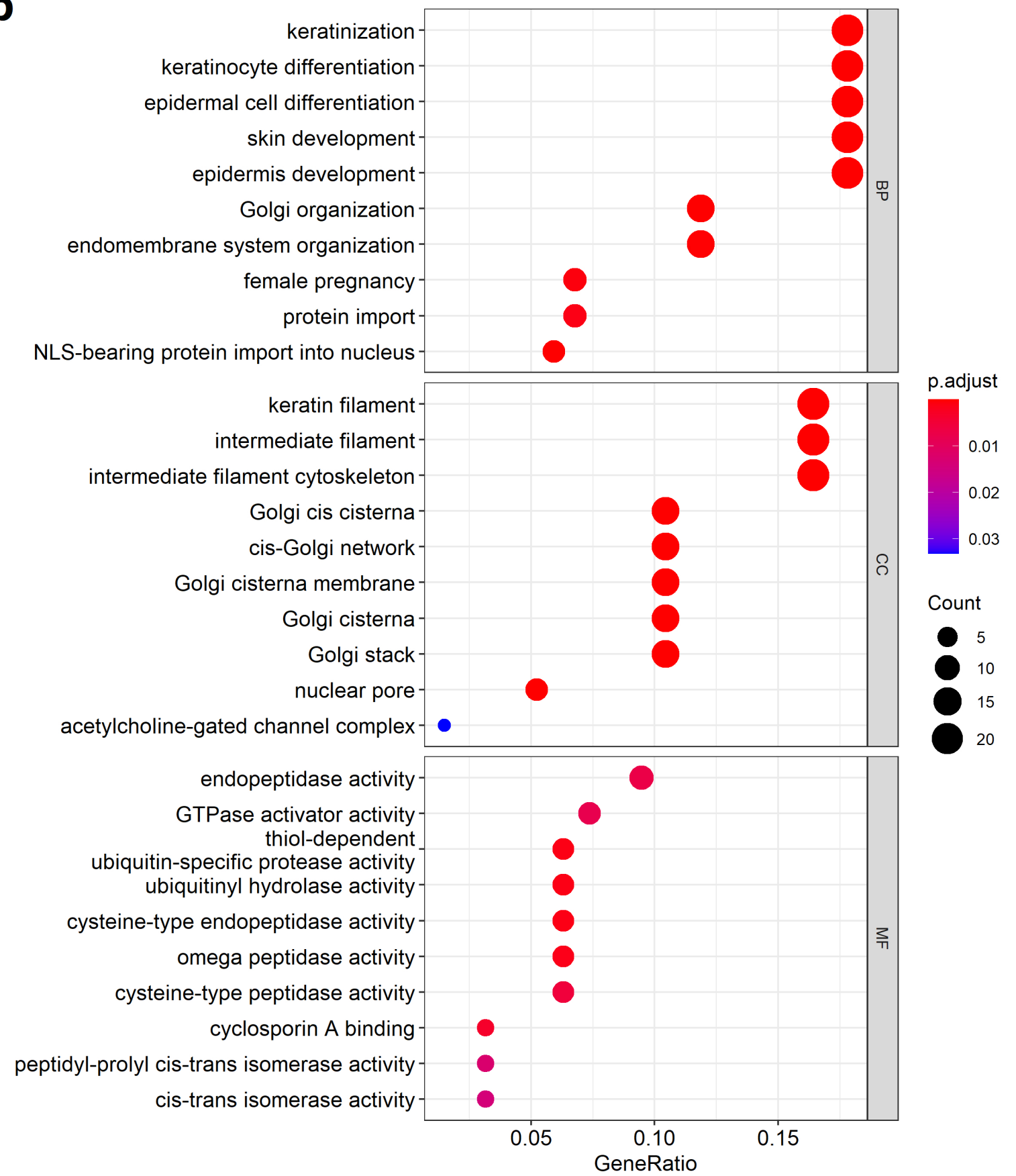

Supplement: Figure S2. Functional enrichment analysis for frequently mutated genes (a) and genes with high-frequency copy number variations (CNVs) based on the Gene Ontology database. BP: biological process; CC: cellular components; MF: molecular function. [file supplementary_figure_2.pdf]

**a**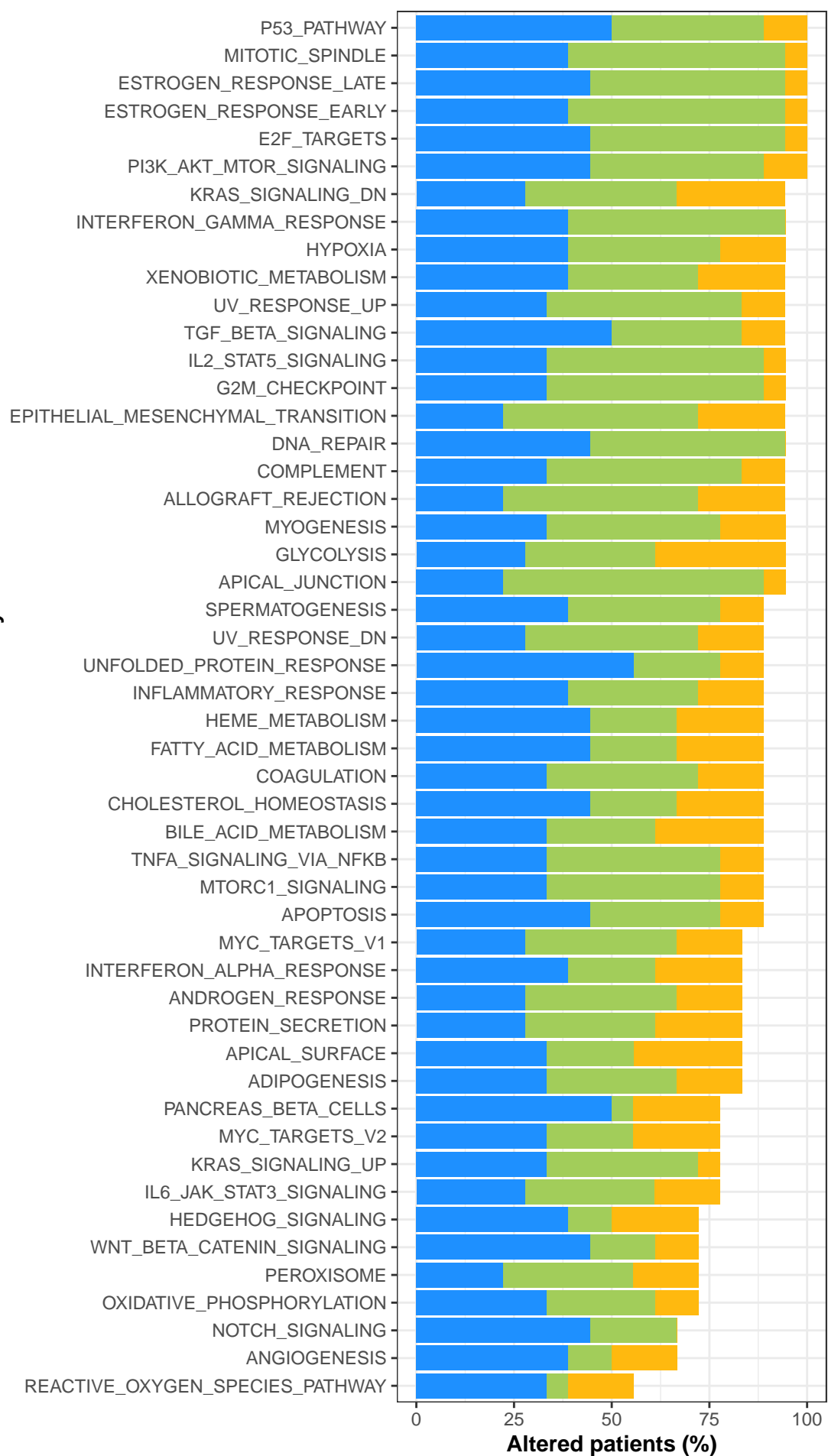**b**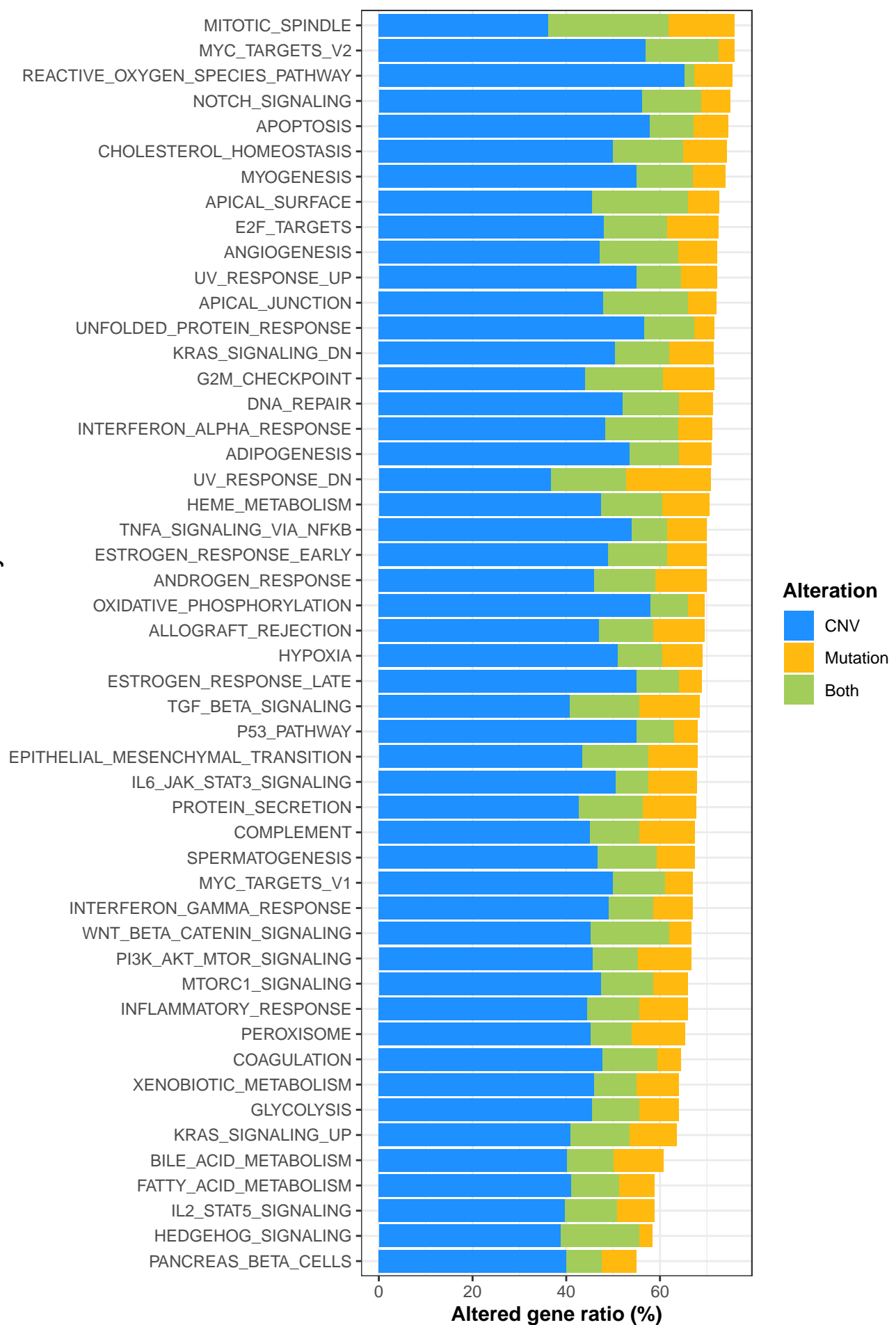

Supplement: Figure S3. The bar plots show the frequency of patients with gene alterations (a), and the altered gene ratio (b) in 50 Hallmark signaling pathways. [file supplementary_figure_3.pdf]

## WNT canonical pathway

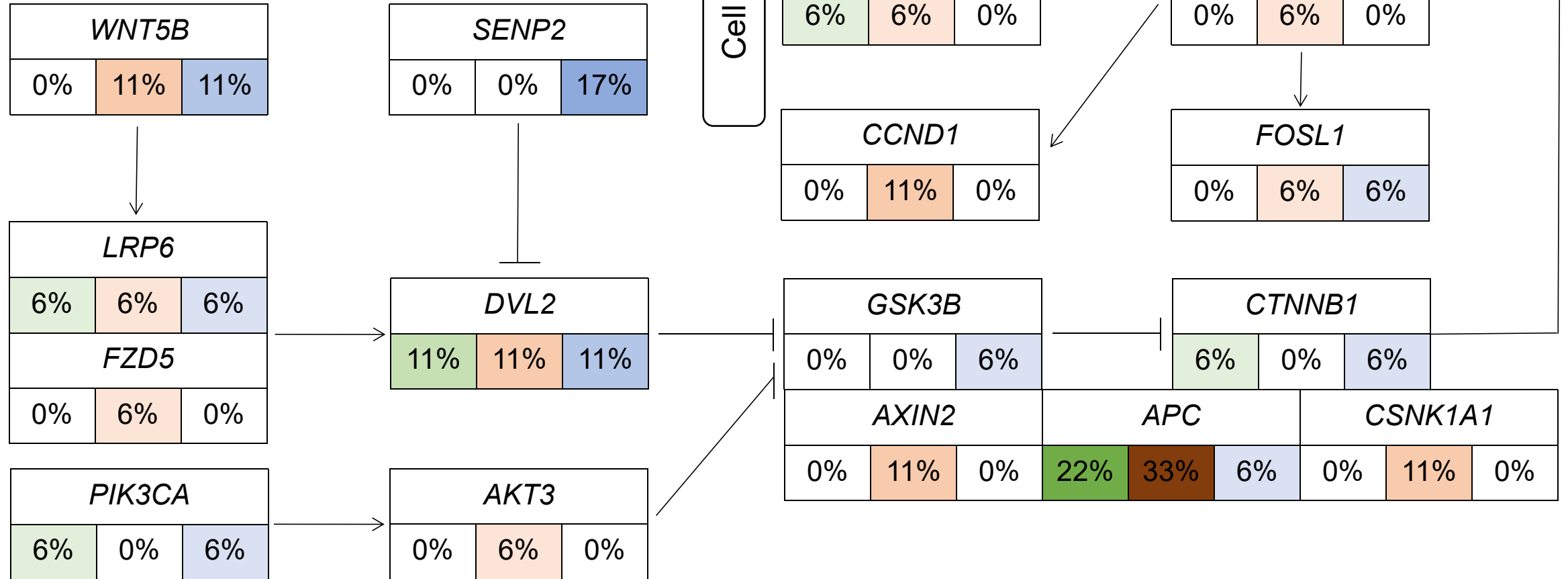

## Pi3k-akt pathway

Supplement: Figure S4. The frequency of somatic mutations and copy number variations are shown for key genes in the WNT canonical signaling pathway based on KEGG. Green represents mutation, red represents copy number amplification, and blue represents copy number deletion. The darker the color, the higher the f [file supplementary_figure_4.pdf]

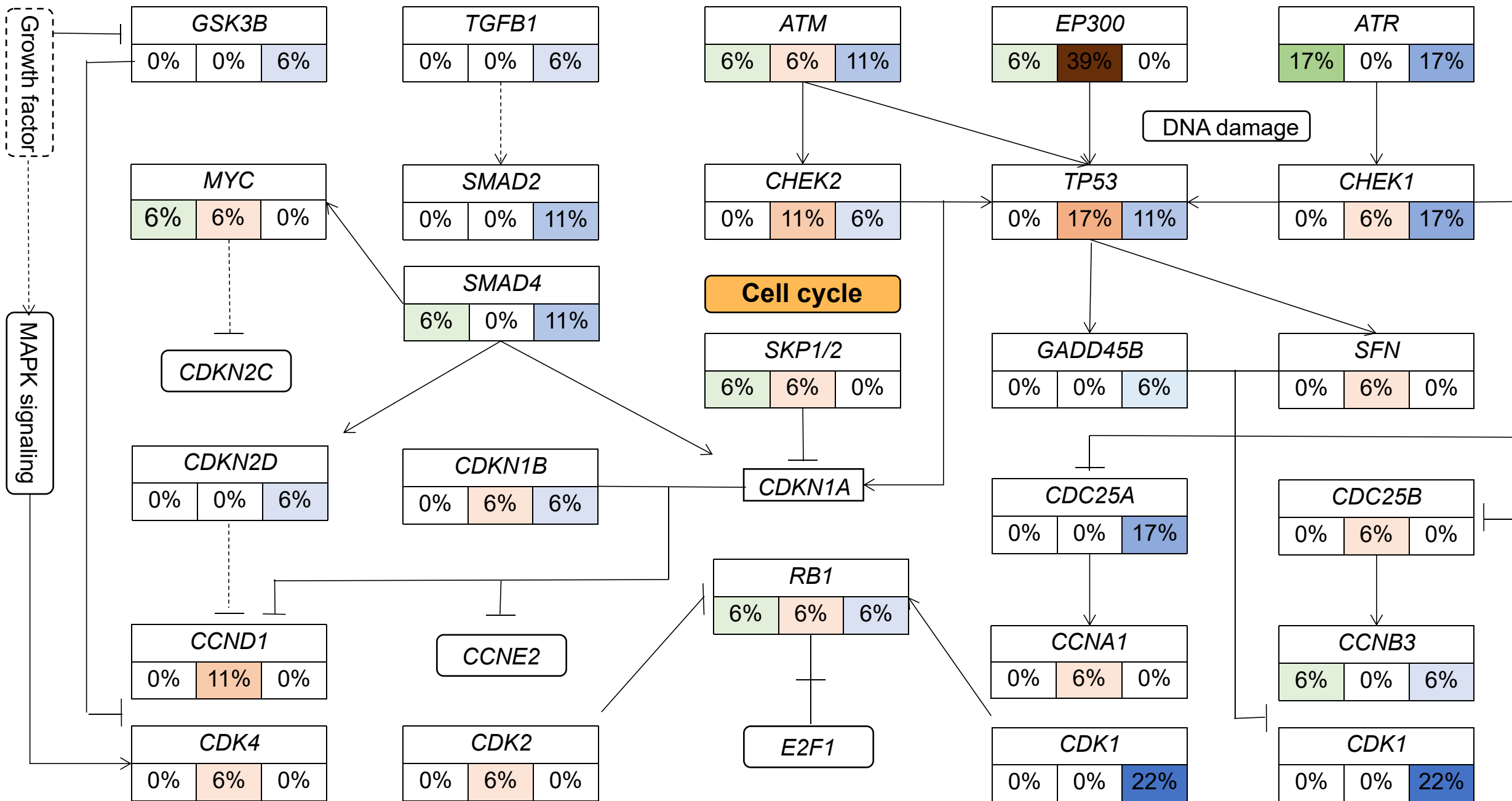

Supplement: Figure S5. The frequency of somatic mutations and copy number variations are shown for key genes in the Cell cycle signaling pathway based on KEGG. Green represents mutation, red represents copy number amplification, and blue represents copy number deletion. The darker the color, the higher the freq [file supplementary_figure_5.pdf]

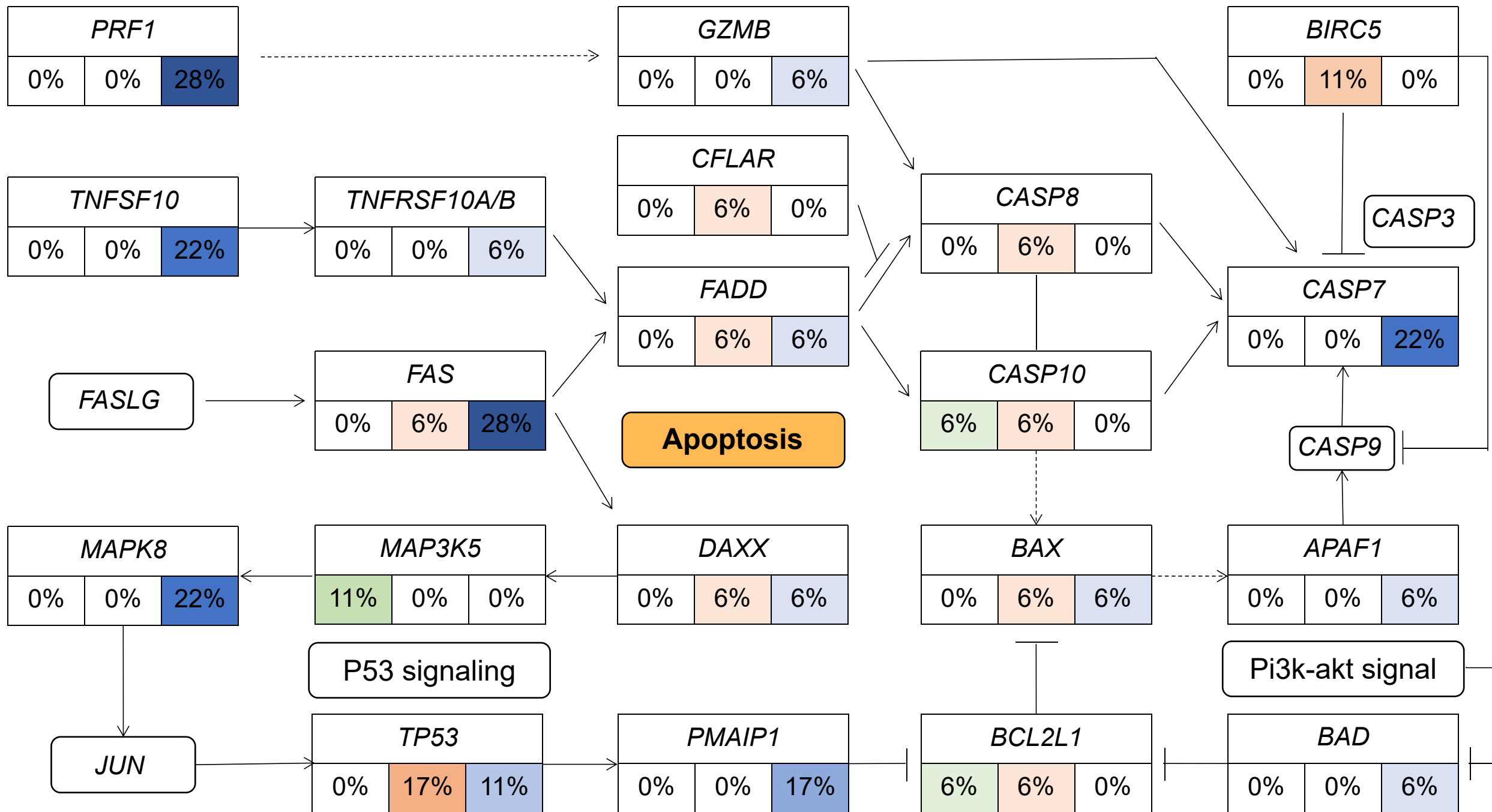

Supplement: Figure S6. The frequency of somatic mutations and copy number variations are shown for key genes in the Apoptosis signaling pathway based on KEGG. Green represents mutation, red represents copy number amplification, and blue represents copy number deletion. The darker the color, the higher the frequ [file supplementary_figure_6.pdf]

## Non-homologous end-joining repair

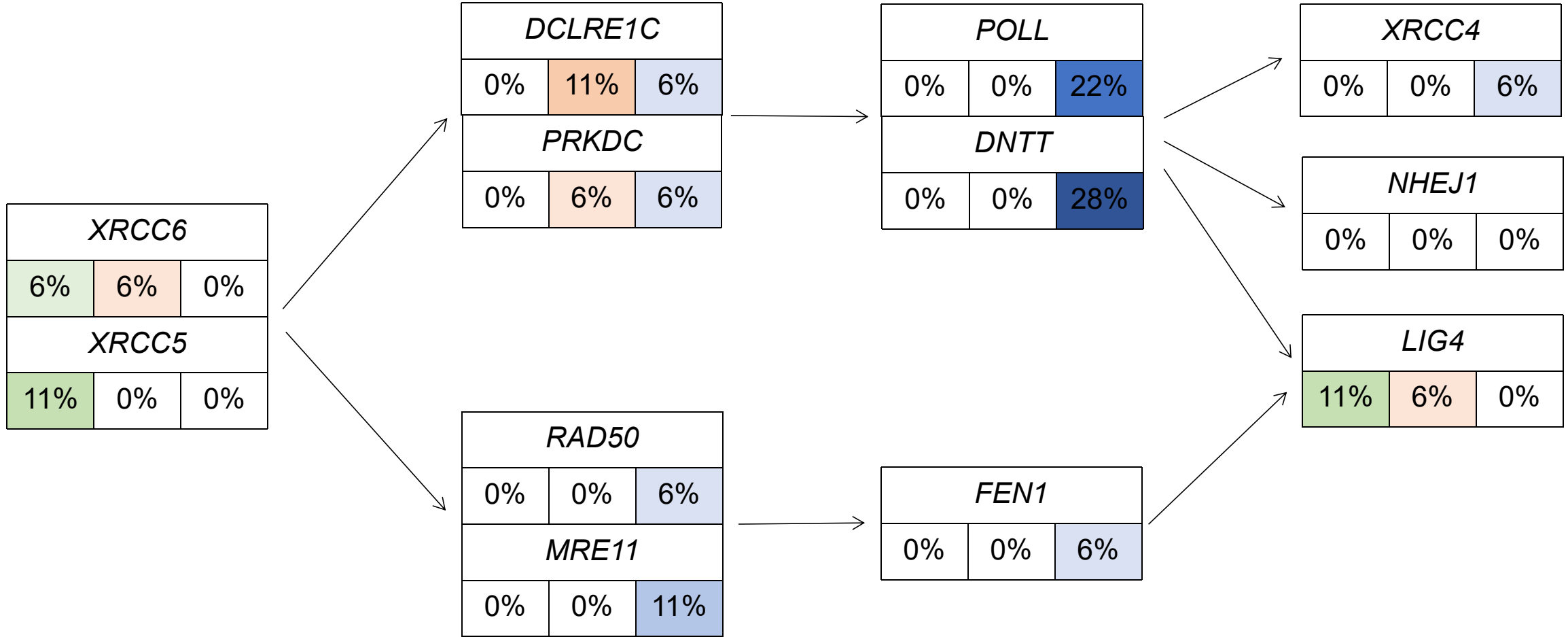

Supplement: Figure S11. The frequency of somatic mutations and copy number variations are shown for key genes in the Non-homologous end-joining repair signaling pathway based on KEGG. Green represents mutation, red represents copy number amplification, and blue represents copy number deletion. The darker the co [file supplementary_figure_11.pdf]

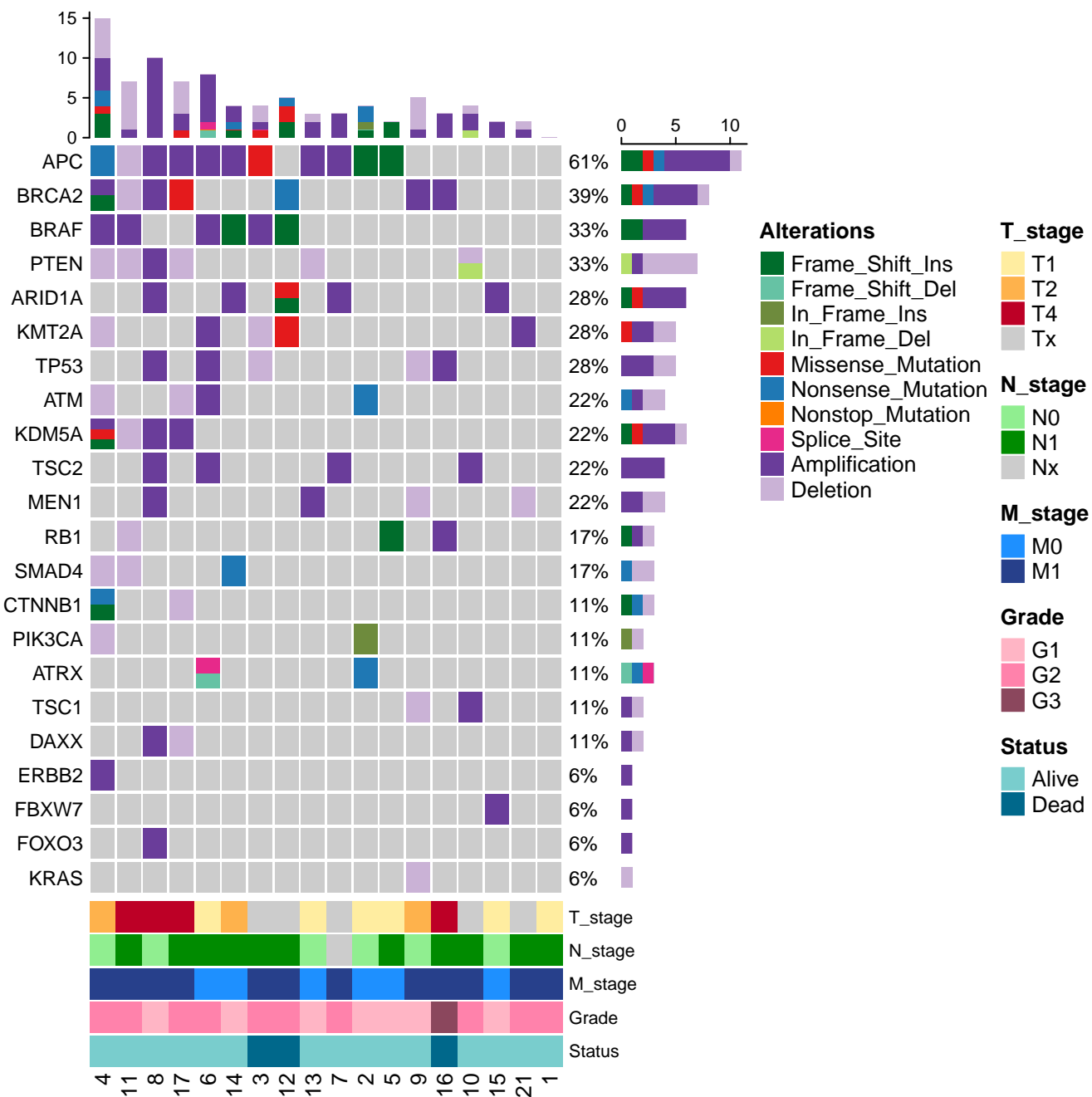

Supplement: Figure S13. The variation frequency and types of common gene variations of neuroendocrine neoplasms (NENs) were reported in previous genomic studies in our rectal NET cohort. [file supplementary_figure_13.pdf]
