# Supplementary material for: Whole-exome sequencing of rectal neuroendocrine tumors
Source: Endocr Relat Cancer. 2023 Aug 2;30(9):e220257. doi: 10.1530/ERC-22-0257 (PMC10450454; doi:10.1530/ERC-22-0257)
Supplement: Figure S7. The frequency of somatic mutations and copy number variations are shown for key genes in the Base excision repair signaling pathway based on KEGG. Green represents mutation, red represents copy number amplification, and blue represents copy number deletion. The darker the color, the highe [file supplementary_figure_7.pdf]

Base excision repair

Short patch BER complex

APEX1			*PARP1*			*LIG3*		
0%	6%	0%	6%	0%	0%	0%	6%	0%
POLB			*XRCC1*					
0%	0%	0%	0%	0%	6%			

Long patch BER complex

POLB			*APEX1*			*FEN1*		
0%	0%	0%	0%	6%	0%	0%	0%	6%
LIG1			*PCNA*			*PARP1*		
6%	0%	6%	0%	0%	0%	6%	0%	0%
			POLD1			*POLE*		
			0%	6%	11%	6%	6%	6%
