# Supplementary material for: Whole-exome sequencing of rectal neuroendocrine tumors
Source: Endocr Relat Cancer. 2023 Aug 2;30(9):e220257. doi: 10.1530/ERC-22-0257 (PMC10450454; doi:10.1530/ERC-22-0257)
Supplement: Figure S8. The frequency of somatic mutations and copy number variations are shown for key genes in the Nucleotide excision repair signaling pathway based on KEGG. Green represents mutation, red represents copy number amplification, and blue represents copy number deletion. The darker the color, the [file supplementary_figure_8.pdf]

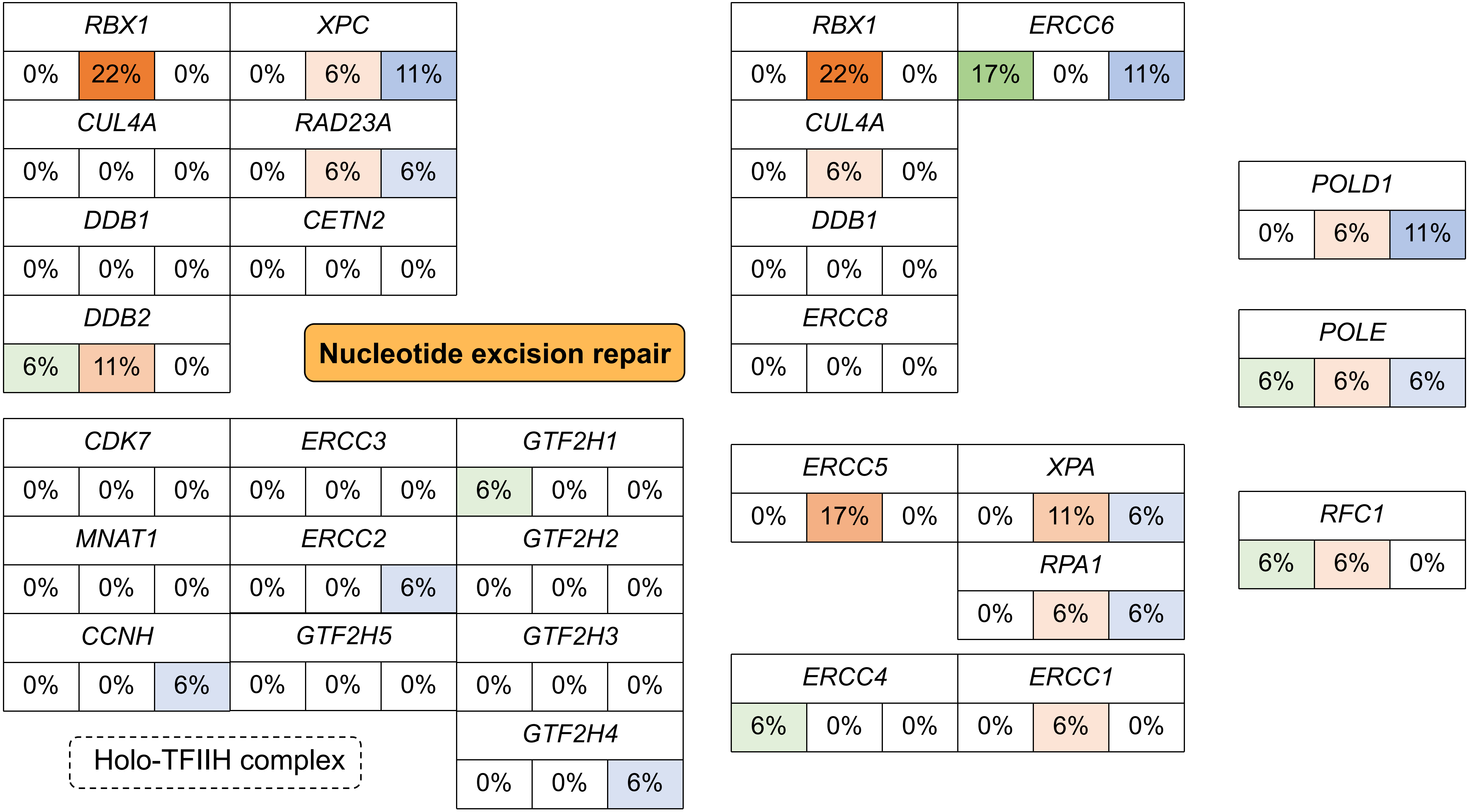

RBX1
0% 22% 0%
XPC
0% 6% 11%
CUL4A
0% 0% 0%
RAD23A
0% 6% 6%
DDB1
0% 0% 0%
CETN2
0% 0% 0%
DDB2
6% 11% 0%
Nucleotide excision repair
RBX1
0% 22% 0%
ERCC6
17% 0% 11%
CUL4A
0% 6% 0%
DDB1
0% 0% 0%
ERCC8
0% 0% 0%
POLD1
0% 6% 11%
POLE
6% 6% 6%
CDK7
0% 0% 0%
ERCC3
0% 0% 0%
GTF2H1
6% 0% 0%
MNAT1
0% 0% 0%
ERCC2
0% 0% 6%
GTF2H2
0% 0% 0%
CCNH
0% 0% 6%
GTF2H5
0% 0% 0%
GTF2H3
0% 0% 0%
GTF2H4
0% 0% 6%
Holo-TFIIH complex
ERCC5
0% 17% 0%
XPA
0% 11% 6%
RPA1
0% 6% 6%
ERCC4
6% 0% 0%
ERCC1
0% 6% 0%
RFC1
6% 6% 0%
