# Supplementary material for: Whole-exome sequencing of rectal neuroendocrine tumors
Source: Endocr Relat Cancer. 2023 Aug 2;30(9):e220257. doi: 10.1530/ERC-22-0257 (PMC10450454; doi:10.1530/ERC-22-0257)
Supplement: Figure S9. The frequency of somatic mutations and copy number variations are shown for key genes in the Mismatch repair signaling pathway based on KEGG. Green represents mutation, red represents copy number amplification, and blue represents copy number deletion. The darker the color, the higher the [file supplementary_figure_9.pdf]

PMS2			MLH1		
0%	11%	0%	0%	28%	17%
MSH6			MSH2		
0%	11%	0%	6%	22%	0%

RFC1		
6%	6%	0%

MLH1			PMS2		
0%	28%	17%	0%	6%	0%
MSH2			MSH3		
6%	22%	0%	0%	6%	6%

RPA1		
0%	6%	6%

POLD1		
0%	6%	11%

LIG1		
6%	0%	6%

MLH1			MLH3		
0%	28%	17%	11%	6%	0%
MSH2			MSH3		
6%	22%	0%	0%	6%	6%

Mismatch repair
