# Supplementary material for: Whole-exome sequencing of rectal neuroendocrine tumors
Source: Endocr Relat Cancer. 2023 Aug 2;30(9):e220257. doi: 10.1530/ERC-22-0257 (PMC10450454; doi:10.1530/ERC-22-0257)
Supplement: Figure S10. The frequency of somatic mutations and copy number variations are shown for key genes in the Homologous recombination repair signaling pathway based on KEGG. Green represents mutation, red represents copy number amplification, and blue represents copy number deletion. The darker the colo [file supplementary_figure_10.pdf]

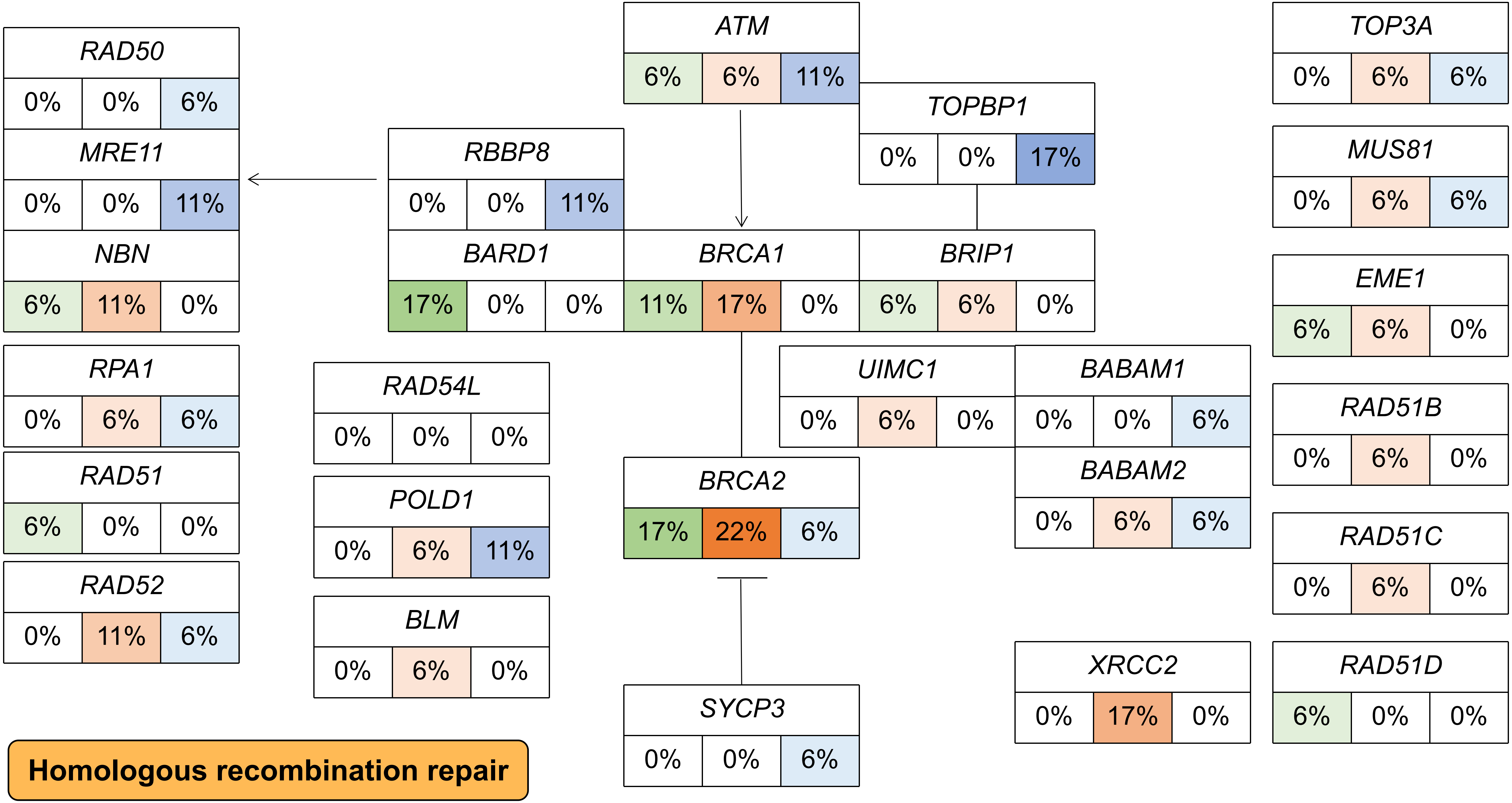
RAD50
0% 0% 6%
MRE11
0% 0% 11%
NBN
6% 11% 0%
RPA1
0% 6% 6%
RAD51
6% 0% 0%
RAD52
0% 11% 6%
RBBP8
0% 0% 11%
ATM
6% 6% 11%
TOPBP1
0% 0% 17%
BARD1
17% 0% 0%
BRCA1
11% 17% 0%
BRIP1
6% 6% 0%
RAD54L
0% 0% 0%
POLD1
0% 6% 11%
BLM
0% 6% 0%
UIMC1
0% 6% 0%
BABAM1
0% 0% 6%
BABAM2
0% 6% 6%
BRCA2
17% 22% 6%
SYCP3
0% 0% 6%
XRCC2
0% 17% 0%
TOP3A
0% 6% 6%
MUS81
0% 6% 6%
EME1
6% 6% 0%
RAD51B
0% 6% 0%
RAD51C
0% 6% 0%
RAD51D
6% 0% 0%
Homologous recombination repair
