# Supplementary material for: Whole-exome sequencing of rectal neuroendocrine tumors
Source: Endocr Relat Cancer. 2023 Aug 2;30(9):e220257. doi: 10.1530/ERC-22-0257 (PMC10450454; doi:10.1530/ERC-22-0257)
Supplement: Figure S12. Gene alterations with significant correlation with overall survival (OS) in the PI3K-AKT pathway and the P53 signaling pathway. Survival analysis indicated that patients with mutation of ITGB8 (P = 0.032) (a), BRCA1 (P = 0.014) (b), SGK1 (P = 0.014) and copy number loss (CNL) of APAF1 (P [file supplementary_figure_12.pdf]

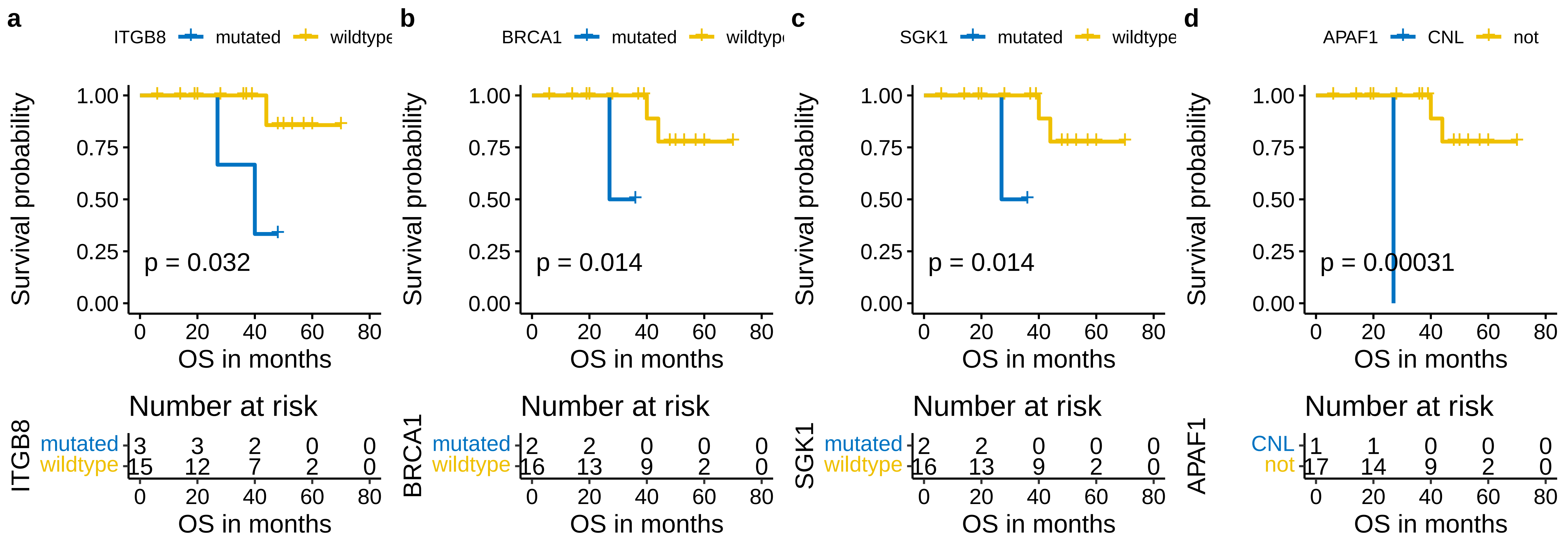

a
ITGB8 mutated wildtype
Survival probability
1.00
0.75
0.50
0.25
0.00
p = 0.032
0 20 40 60 80
OS in months
Number at risk
ITGB8
mutated 3 3 2 0 0
wildtype 15 12 7 2 0
OS in months
b
BRCA1 mutated wildtype
p = 0.014
BRCA1
mutated 2 2 0 0 0
wildtype 16 13 9 2 0
c
SGK1 mutated wildtype
p = 0.014
SGK1
mutated 2 2 0 0 0
wildtype 16 13 9 2 0
d
APAF1 CNL not
p = 0.00031
APAF1
CNL 1 1 0 0 0
not 17 14 9 2 0
